# Extracellular Vesicles Derived from SIPA1^high^ Breast Cancer Cells Enhance Macrophage Infiltration and Cancer Metastasis through Myosin-9

**DOI:** 10.3390/biology11040543

**Published:** 2022-03-31

**Authors:** Lingyun Feng, Jun Weng, Chenguang Yao, Ruyuan Wang, Ning Wang, Yilei Zhang, Yoshimasa Tanaka, Li Su

**Affiliations:** 1 Key Laboratory of Molecular Biophysics of Ministry of Education, College of Life Science and Technology, Huazhong University of Science and Technology, Wuhan 430074, China; lingyun_feng@hust.edu.cn (L.F.); jweng@hust.edu.cn (J.W.); d201780472@hust.edu.cn (C.Y.); wangruyuan@hust.edu.cn (R.W.); wangning@hust.edu.cn (N.W.); 2 The Institute of Molecular and Translational Medicine, Department of Biochemistry and Molecular Biology, School of Basic Medical Sciences, Xi’an Jiaotong University Health Science Center, Xi’an 710061, China; zhangyilei@xjtu.edu.cn; 3 Center for Medical Innovation, Nagasaki University, 1-7-1, Sakamoto, Nagasaki 852-8588, Japan

**Keywords:** breast cancer, extracellular vesicles, macrophage, myosin-9, SIPA1

## Abstract

**Simple Summary:**

The high expression of signal-induced proliferation-associated 1 (SIPA1) in breast cancer could aggravate cancer cell metastasis, but how the tumour microenvironment is involved in this incident is unknown. In this study, we investigated whether breast cancer cells with high SIPA1 expression recruited macrophages into the tumour microenvironment. We also found that extracellular vesicles (EVs) derived from MDA-MB-231 cells significantly enhanced macrophage migration, compared with that from *SIPA1*-knockdown MDA-MB-231 cells both in vitro and in vivo. In terms of the mechanism, SIPA1 in cancer cells modulated the key protein myosin-9 in EVs and promoted macrophage infiltration via EVs. We confirmed that either down-regulating SIPA1 expression or blocking myosin-9 by its inhibitor, blebbistatin, led to the suppression of macrophage infiltration. These findings contribute to a deep understanding of how SIPA1 regulates the tumour microenvironment in breast cancer to facilitate tumour metastasis and provide a basis for the development of therapeutics against breast cancer metastasis.

**Abstract:**

Tumour cell metastasis can be genetically regulated by proteins contained in cancer cell-derived extracellular vesicles (EVs) released to the tumour microenvironment. Here, we found that the number of infiltrated macrophages was positively correlated with the expression of signal-induced proliferation-associated 1 (SIPA1) in invasive breast ductal carcinoma tissues and MDA-MB-231 xenograft tumours. EVs derived from MDA-MB-231 cells (231-EVs) significantly enhanced macrophage migration, compared with that from *SIPA1*-knockdown MDA-MB-231 cells (231/si-EVs) both in vitro and in vivo. We revealed that SIPA1 promoted the transcription of *MYH9*, which encodes myosin-9, and up-regulated the expression level of myosin-9 in breast cancer cells and their EVs. We also found that blocking myosin-9 by either down-regulating SIPA1 expression or blebbistatin treatment led to the suppression of macrophage infiltration. Survival analysis showed that breast cancer patients with high expression of *SIPA1* and *MYH9* molecules had worse relapse-free survival (*p* = 0.028). In summary, SIPA1^high^ breast cancer can enhance macrophage infiltration through EVs enriched with myosin-9, which might aggravate the malignancy of breast cancer.

## 1. Introduction

Extracellular vesicles (EVs) are nanoscale lipid bilayer-enclosed particles in extracellular spaces secreted from numerous cell types [1,2]. Tumour cell-derived EVs play pivotal roles in the intercellular communication that occurs in the tumour microenvironment, promoting angiogenesis and metastasis [3,4], exhibiting immunomodulatory effects [5,6], and remodelling surrounding parenchymal tissues [7,8].

Breast cancer metastasis, which accounts for 90% of cancer deaths, is a multi-stage process culminating in colonisation in a remote environment [9,10]. EVs secreted by breast cancer cells contain tumour antigens and immunosuppressive factors and can inhibit tumour antigen-specific immune responses by regulating the function of macrophages and dendritic cells [11,12].

Tumour-associated macrophages (TAMs) are involved in the migration, invasion, and metastasis of tumour cells [13], and bring about the development of various malignant solid tumours, such as breast cancer [14]. Linde et al. demonstrated that subpopulations of cancer cells require macrophages for pre-dissemination and metastasis [15]. Pulmonary metastases were not observed in macrophage-deficient mice [16]. Hoshino et al. reported that exosomes expressing ITGαvβ5 specifically bound to Kupffer cells and mediated liver tropism [17]. Tumour-secreted EVs contain proteins, nucleic acids, and lipids, which contributes to intercellular communication [18,19]. However, it is not clear which molecules are mainly involved in breast cancer metastasis.

Signal-induced proliferation-associated 1 (SIPA1) is markedly expressed in MDA-MB-231, a highly metastatic human breast cancer cell line [20]. SIPA1 could be a key factor for tumour metastasis and recurrence in breast [21], colorectal [22], prostate [23], and lung cancer [24]. SIPA1 is involved in many aspects of malignancy, such as breast cancer cell metastasis [20,25], maintenance of stemness, and drug resistance [26]. Recent studies have reported that SIPA1 locates in the nucleus of breast cancer cells, and could interact with the promoter of genes, such as *ITGB1*, *CD44*, and *EPAS1*, and upregulate their transcription [20,26,27,28]. In this study, we set out to clarify the effect of high expression of SIPA1 in breast cancer cells on macrophage recruitment in the tumour microenvironment. We demonstrated that SIPA1 could interact with the *MYH9* promoter, increasing myosin-9 accumulation in EVs. Moreover, SIPA1 increased macrophage infiltration and led to cancer metastasis via myosin-9-enriched EVs. These results suggest that the SIPA1-regulated EVs from cancer cells may be an important regulator of cancer metastasis.

## 2. Materials and Methods

### 2.1. Cell Lines and Breast Cancer Tissues

Human cell lines MDA-MB-231, MCF7, THP1, and HEK293T were purchased from the China Center for Type Culture Collection (Wuhan, China). BT549 and RAW264.7 were purchased from Procell (Wuhan, China). Cells were maintained following the previous study [27,29,30]. Breast cancer tissue microarray (TMA) slides (HBreD090PG01), including 65 cases of invasive ductal carcinoma, were provided by Shanghai Outdo Biotech Co., Ltd (Shanghai, China). (Ethics approval number: SHYJS-CP-1910006).

### 2.2. Plasmid and Cell Line Construction

The plasmid pcDNA3-*SIPA1* was constructed as previously described [20]. *SIPA1* and *MYH9* shRNA sequences are provided in Table S1. pLKO.1-GFP shRNA constructs for the knockdown of *SIPA1* and *MYH9* were prepared as lentivirus. Plasmids of psPAX2, pMD2.G, and pRL-TK were purchased from Addgene (Watertown, MA, USA). The stable knockdown cell lines MDA-MB-231/sh-*SIPA1* (abbreviated as 231/si), BT549/sh-*SIPA1*, and MDA-MB-231/sh-*MYH9* (abbreviated as 231/sh-*MYH9*) were established following the previous study [28]. An *SIPA1*-overexpressing MCF7 cell line (MCF7/*SIPA1*) was established as previously described [26]. The expression plasmid pCMV7.1-*MYH9* (MiaoLingBio, Wuhan, China) was transfected into 231/si cells using Lipofectamine 2000 (Thermo Fisher Scientific, Waltham, MA, USA). The plasmid of pGL4-*MYH9* containing a −1000/+313-bp *MYH9* promoter region was constructed as previously described [31].

### 2.3. Animal Experiments

Animal experiments were processed following the guidelines of Laboratory Animal Care formulated by the National Society of Medical Research and those for the US National Institutes of Health. The protocol was approved by the Animal Care and Use Committee of Huazhong University of Science and Technology (Ethic Code: S797).

The MDA-MB-231 and 231/si cell subcutaneous xenograft mouse models were established as previously described [26]. The orthotopic MDA-MB-231 tumour model for EV treatments was established as below. MDA-MB-231 cells (5 × 10^6^) were injected into the fourth mammary fat pad of 5-week-old female specific-pathogen-free BALB/c nude mice (Liaoning Changsheng Biotechnology Co., Ltd., Shenyang, China), and tumour-bearing mice were grouped randomly. From day 5 post tumour inoculation, EVs were injected intratumorally (25 μg/tumour) every 2 days [32]. The tumour size was measured by using a digital calliper and the volume was calculated using the formula volume (mm^3^) = [width (mm)]^2^ × [length (mm)]/2 [33]. On day 20, the MDA-MB-231 tumours were carefully excised with scissors and the skin was sutured to maintain the animals for an additional 2 weeks. The mice were sacrificed on day 35. Lung tissues were fixed with Bouin’s fluid, and the numbers of metastatic tumour colonies were counted [34]. After fixation, tissue slides were stained with haematoxylin and eosin (H&E).

### 2.4. Isolation and Characterisation of EVs

EVs were isolated following a previously described sequential ultracentrifugation method [35]. Briefly, breast cancer cells plated in 10 mm cell culture dishes were incubated in the serum-free RPMI1640 medium for 24 h at 37 °C with 5% CO_2_. The culture supernatant was collected and centrifuged at 500× *g* for 10 min, and then at 2000× *g* for 30 min to remove cell debris. Next, the supernatant was filtered through a 0.22 μm Millipore membrane and centrifuged at 100,000× *g* for 120 min to collect the pellet. The pellet was washed in ice-cold phosphate-buffered saline (PBS) and subjected to ultracentrifugation at 100,000 × *g* for 120 min. The centrifugation process was performed at 4 °C. Finally, the pellets (EVs fraction) were re-suspended in 100 µL PBS. The EVs were stored at −80 °C and used within 2 weeks. The protein concentration in the EVs was measured using the BCA protein assay kit (Thermo). The quality of the EVs was confirmed by imaging with a HITACHI HT770 transmission electron microscope (TEM) (HITACHI, Tokyo, Japan) and conducting nanoparticle tracking analysis (NTA) with a ZetaView PMX-120 video microscope (Particle Metrix, Meerbusch, Germany) and ZETAVIEW software [36]. The expression of the EV typical marker proteins CD63, TSG101, Alix, and syntenin-1 were determined by Western blotting, in which syntenin-1 was used as the internal control [36].

### 2.5. Mass Spectrometry

Proteomics analysis of EV samples was processed on a liquid chromatography electrospray ionisation tandem mass spectrometer (LC-ESI MS/MS, Shanghai Applied Protein Technology Co., Ltd., Shanghai, China) as previously reported [37]. After EVs (equivalent to 30 μg total proteins) were treated with trypsin digestion, the peptides were collected and loaded onto the Easy-nLC1000 system equipped with the orbitrap Q Exactive MS (Thermo). The LC-ESI MS/MS full-scan signals were acquired within the range of the precursor ion from 300 to 1800 m/z, and original files were imported into Mascot software (version 2.2, Matrix Science, London, UK) for protein candidate identification and characterisation. The reference database was uniprot_Homo_sapiens_173343_20191014.fasta (173,343 protein sequences, download date of 14 October 2019).

### 2.6. Immunofluorescence Staining and Immunohistochemistry

Immunofluorescence staining was performed on TMA sections [38]. The primary and secondary antibodies are listed in Table S2. Nuclei were counterstained with 4′,6-diamidino-2-phenylindole (200 ng/mL, Sigma Aldrich, St. Louis, MO, USA). The TMA was visualised using a digital slide scanner (Pannoramic P250, 3DHISTECH, Budapest, Hungary) and at least three fields per sample were acquired from randomly selected fields of view. The fluorescence images were analysed using ImageJ software [39]. For immunohistochemistry, the sections were incubated with the primary antibodies at 4 °C overnight, and then with secondary antibodies at 25 °C for 1 h. The signals were visualised by a DAB kit (Servicebio, Wuhan, China) and counterstained with haematoxylin (Servicebio). The antibodies are listed in Table S2. Images were acquired using StrataFAXS (TissueGnostics, Vienna, Austria); the percentage of positive cells was determined using StrataQuest software (TissueGnostics) [40].

### 2.7. Western Blotting Analysis and Quantitative Real-Time PCR (qRT-PCR)

Western blotting analysis was performed after cell lysates and EV samples were prepared in RIPA buffer as previously described [41]. The signals were visualised using an ECL Western blotting substrate (Thermo). The antibodies are listed in Table S2. qRT-PCR was conducted as previously described [42] and the primers are listed in Table S1. The relative expression of each gene was calculated using the 2^−ΔΔCT^ method from triplicate reactions, in which GAPDH was used for normalisation.

### 2.8. Chromatin Immunoprecipitation Assays

Chromatin immunoprecipitation (ChIP) assays were performed as previously described [43]. Briefly, protein-DNA samples of MDA-MB-231 were cross-linked with formaldehyde treatment (1.42% final concentration) and treated with an anti-SIPA1 antibody (Table S2). The SIPA1-binding DNA was purified using a DNA recovery kit (Tiangen Biotech, Beijing, China); the *MYH9* promoter fragment was amplified through PCR using the specific primers (Table S1).

### 2.9. Dual-Luciferase Reporter Gene Assay

Reporter assays were performed using HEK293T cells transfected with the indicated plasmids and analysed using a Dual-Luciferase Reporter Assay kit (Promega, Madison, WI, USA). The luciferase activity was measured by a GloMax 20/20 luminometer (Promega). The expression levels were normalised with respect to those for cells co-transfected with pRL-TK plasmid.

### 2.10. Cell Migration Assay

THP1 cells were treated with 10 ng/mL phorbol 12-myristate 13-acetate (PMA, Beijing Solarbio Science & Technology Co., Ltd., Beijing, China) to induce the differentiation of the cells to macrophages [44]. The THP1-derived macrophages (1 × 10^4^ cells) were seeded onto an upper chamber of an 8 μm-pore transwell 24-well plate (BD Biosciences, NJ, USA), and supernatants or EVs (200 μg/mL) derived from different cells were added to the lower chamber. After incubation at 37 °C with 5% CO_2_ for 24 h, the THP1-derived macrophages that migrated across the membranes were fixed with methanol, stained with 0.1% crystal violet solution, and counted under a Nikon 80i microscope (Minato-ku, Tokyo, Japan) [45].

### 2.11. EV Uptake Assay

EVs were labelled using Dil red dye (Beyotime, Shanghai, China) following the instructions. Briefly, EVs were incubated with Dil (1:500 dilutions in PBS) at 4 °C for 30 min and isolated using the Total Exosome Isolation kit (Invitrogen, Carlsbad, CA, USA). After being incubated with Dil-labeled EVs at 37 °C for 24 h, the macrophages were imaged using an Olympus FV1000 laser confocal microscope (Olympus, Tokyo, Japan).

### 2.12. Bioinformatic Analysis

The tumour and immune system interactions database (TISIDB) (http://cis.hku.hk/TISIDB/, accessed on 28 January 2022) was used to deduce the functions of candidate genes, especially their roles in tumour–immune system interactions through high-throughput data analysis and literature mining [46]. Differentially expressed protein candidates were obtained by comparing two EVs datasets (231-EVs vs. 231/si-EVs), and gene ontology (GO) annotation and enrichment analysis was carried out using Metascape 3.5 (http://metascape.org/, accessed on 8 December 2020) to identify functional candidates [47]. A Venn diagram was drawn using an online Venn tool (https://bioinfogp.cnb.csic.es/tools/venny/index.html, accessed on 10 December 2020) [48]. The mRNA expressional correlation between *SIPA1* and *MYH9* in the breast-invasive carcinoma (TCGA, Firehose Legacy) study (n = 1100) was surveyed using the cBioPortal platform (www.cbioportal.org, accessed on 24 January 2022) [49,50]. Kaplan–Meier survival analysis of both breast cancer (n = 4929) and TNBC (n = 392) was performed using publicly available gene chip datasets in the Kaplan–Meier (KM) plotter (www.kmplot.com, accessed on 29 January 2022) [51]. The patients were divided into high vs. low expressors based on the median value for *MYH9* gene.

### 2.13. Statistical Analyses

Statistical analyses were carried out using Graphpad Prism (version 8.0, San Diego, CA, USA) software. The two-tailed Student’s *t*-test was used for comparison between treatment and control groups. For multiple comparisons, the one-way ANOVA plus two-sided Tukey test was applied. Correlation analyses were performed using the Spearman correlation test. All values are expressed as the mean ± SD unless otherwise indicated, and *p* < 0.05 was considered significant. * *p* < 0.05; ** *p* < 0.01; *** *p* < 0.001; **** *p* < 0.0001; ns, not significant (*p* > 0.05).

## 3. Results

### 3.1. SIPA1-Expressing Breast Cancer Cells Recruit Macrophages into the Tumour Milieu

**Figure 1:**
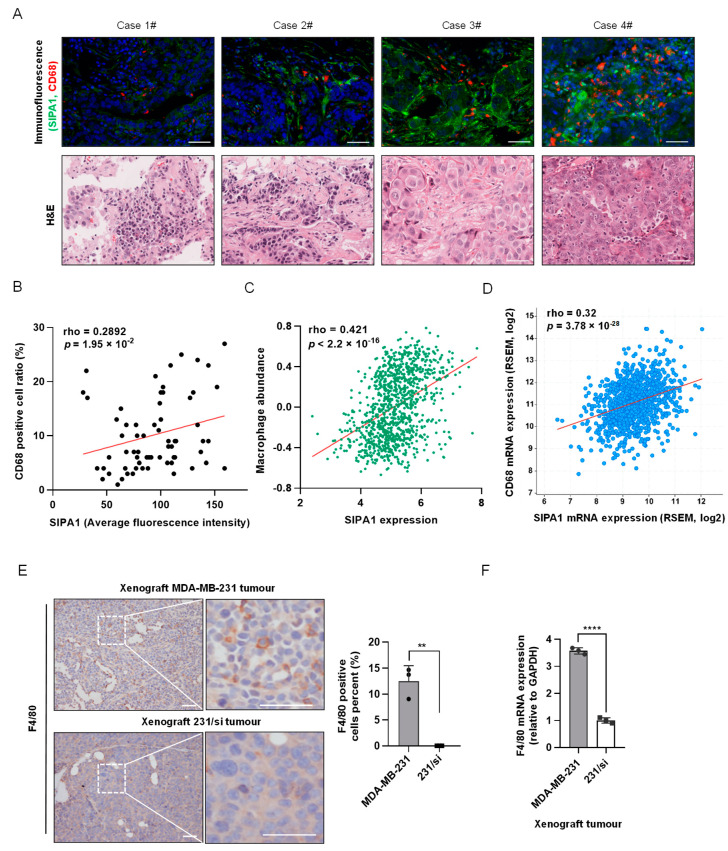
Correlation between SIPA1 expression level in breast cancer tissues and macrophage abundance in tumour microenvironment. (**A**) Immunohistochemical staining of CD68 (red), SIPA1 (green), and 4′,6-diamidino-2-phenylindole (blue) of the representative breast cancer tissues (upper panels), along with the collative haematoxylin and eosin images (lower panels). Case 1# to Case3#: Grade II; Case 4#: Grade III. Scale bar: 50 μm. (**B**) The correlation between the percentage of CD68-positive cells (positive cells/total cells, %) and average fluorescence intensity of SIPA1 in tissue microarray (n = 65). (**C**) The correlation between *SIPA1* expression and macrophage abundance based on the TISIDB database. (**D**) Co-expression analysis of *SIPA1* and *CD68* in breast cancer via cBioPortal platform (n = 1100). (**E**) Immunohistochemical analysis (left panel) and quantitative analysis (right panel) of the macrophage marker F4/80 in MDA-MB-231 or 231/si cell xenograft tumours. Scale bar: 50 μm; ** *p* < 0.01. (**F**) Determination of F4/80 mRNA levels, normalised to GAPDH, in MDA-MB-231 or *SIPA1*-knockdown MDA-MB-231 (231/si) cell xenograft tumours by quantitative real-time PCR (qRT-PCR, n = 3); **** *p* < 0.0001.

To analyse the correlation between the expression level of SIPA1 in breast cancer cells and the macrophages that infiltrated the tumour microenvironment, we examined the TMA slide with breast cancer samples for the expression of SIPA1 and that of CD68, a human macrophage-specific marker (Figure 1A, upper panels) with H&E staining (Figure 1A, lower panels). The macrophages markedly infiltrated the breast cancer tissues expressing a high level of SIPA1, whereas only a few macrophages were observed in the tissues expressing a low level of SIPA1. The quantitative analysis of the average fluorescence intensity of SIPA1 and the ratio of CD68-positive cells in the tumour milieu revealed that the expression level of SIPA1 in breast cancer positively correlated with the ratio of infiltrated macrophages in invasive ductal carcinoma samples (Figure 1B). By analysing the transcriptome data of 1100 Breast Invasive Carcinoma (BRCA) samples in The Cancer Genome Atlas Program (TCGA) database, we found that the mRNA expression level of *SIPA1* positively correlated with macrophage abundance and pan-macrophage marker *CD68* (Figure 1C,D). MDA-MB-231 cells express a high level of SIPA1 and MCF7 cells express a marginal level of SIPA1 (Figure S1A, left panel), which was consistent with the reports [20,28]. Next, we established a 231/si cell line with stable knockdown of *SIPA1* in the MDA-MB-231 cell (Figure S1A, right panel). Hence, we established parental MDA-MB-231 cells and 231/si cells in xenograft mouse models. As shown in Figure 1E, we visualised the pan-macrophage marker F4/80, a mouse macrophage marker, in the tumour microenvironment and found that more macrophages infiltrated the tumour milieu in the MDA-MB-231 xenograft tumour than those in the 231/si xenograft tumour. The mRNA expression level of F4/80 in the 231/si xenograft tumour was much lower than that in the parental MDA-MB-231 xenograft tumour (Figure 1F). Moreover, the M1 macrophage marker CD86 and the M2 macrophage marker molecule CD206 in the MDA-MB-231 xenograft tumour were higher than those in the 231/si xenograft tumour (Figure S1B). It was most likely that breast cancer cells with a high level of SIPA1 expression recruited macrophages into the tumour microenvironment.

### 3.2. EVs Derived from SIPA1-Expressing Breast Cancer Cells Promote Macrophage Migration

**Figure 2:**
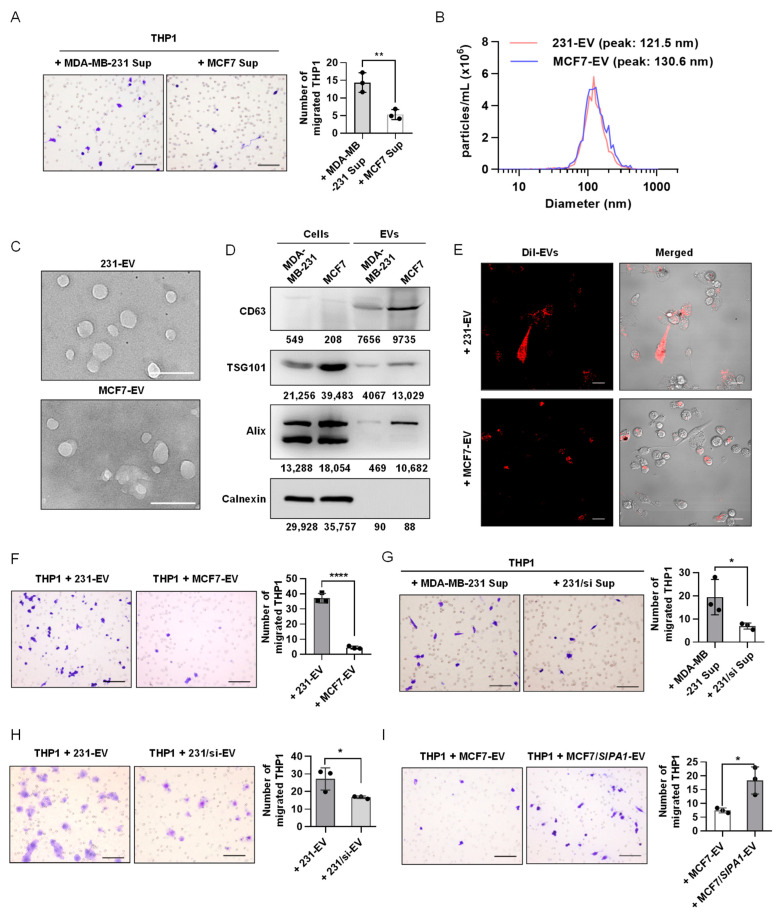
Extracellular vesicles (EVs) derived from SIPA1^high^ breast cancer cells enhance macrophage migration. (**A**) Representative images (left panel) and quantitative analysis (right panel, n = 3) of the migrated macrophages treated with the culture supernatant (Sup) of MCF7 or MDA-MB-231 cells by transwell assay. Scale bar: 100 μm; ** *p* < 0.01. (**B**) The diameter measurement of EVs by nanoparticle tracking analysis. (**C**) Representative transmission electron microscopy images of 231-EVs and MCF7-EVs. Scale bar: 200 nm. (**D**) Detection of CD63, TSG101, Alix, and calnexin by Western blotting in whole-cell and EV lysates. (**E**) Confocal microscopic imaging of the internalisation of EVs into THP1-derived macrophages. The fluorescence of Dil-stained EVs shown in red. Scale bar: 20 μm. (**F**) Representative images (upper panel) and quantitative analysis (lower panel, n = 3) of the migrated macrophages treated with the EVs (200 μg/mL) isolated from MCF7 and MDA-MB-231 cells by transwell assay. Scale bar: 100 μm; **** *p* < 0.0001. (**G**) Representative images (left panel) and quantitative analysis (right panel, n = 3) of the migration of THP1-derived macrophages after the treatment with MDA-MB-231- or 231/si-derived supernatants. Scale bar: 100 μm; * *p* < 0.05. (**H**) Representative images (left panel) and quantitative analysis (right panel, n = 3) of the migration of THP1-derived macrophages after the treatment with the EVs (200 μg/mL) derived from MDA-MB-231 and 231/si cells, respectively. Scale bar: 100 μm; * *p* < 0.05. (**I**) Representative images (left panel) and quantitative analysis (right panel, n = 3) of the migration of THP1-derived macrophages after the treatment with the EVs (200 μg/mL) derived from MCF7 and MCF7/*SIPA1* cells, respectively. Scale bar: 100 μm; * *p* < 0.05.

To detect the effect of the breast cancer cells with variable SIPA1-expressing levels on macrophage infiltration, we first examined the effect of culture supernatants on the migration of macrophages. The supernatants of MDA-MB-231 and MCF7 cells were collected and treated on PMA-differentiated THP1 macrophages. The number of THP1-derived macrophages transmigrated through the membrane into the wells supplemented with the MDA-MB-231 culture supernatant was significantly higher than that with the culture supernatant of MCF7 shown in Figure 2A. Next, we isolated EVs from the culture supernatants of MDA-MB-231 and MCF7, following the procedures illustrated in Figure S2A. NTA revealed that the size of MDA-MB-231 cell-derived EVs (231-EVs) and MCF7 cell-derived EVs (MCF7-EVs) mainly ranged from 30–200 nm, with an average size of 132.5 and 138.1 nm, respectively (Figure 2B). TEM images showed that the size and morphology of the vesicles were characteristic of conventional EVs (Figure 2C). Consistent with the physicochemical properties [52,53], three EV marker proteins, CD63, TSG101, and Alix were all detected positively in breast cancer cell-derived vesicles through Western blotting analysis, whereas calnexin as a negative EV marker was not detected in the EV fractions (Figure 2D). These vesicles prepared from breast cancer cells were consistent with the EV definition in the guidelines by the International Society of Extracellular Vesicles [1]. Moreover, we examined the effect of EVs derived from breast cancer cells on the migration of macrophages. When THP1-derived macrophages were treated with Dil-labelled EVs, they were efficiently engulfed by the macrophages (Figure 2E). It is noteworthy that 231-EVs significantly promoted the migration of THP1-derived macrophages at a concentration of 200 μg/mL (Figure S2B). Then, we compared the migrating capabilities of macrophages by a transwell assay after the cells were stimulated with 231-EVs and MCF7-EVs, respectively. The results revealed that the migrated macrophages treated with EVs of SIPA1 high-expressing MDA-MB-231 are much more than those treated with EVs of SIPA1 low-expressing MCF7 (Figure 2F).

To investigate whether high-expressing SIPA1 in breast cancer cells is involved with the migration of macrophages, we treated THP1-derived macrophages with MDA-MB-231 culture supernatant as well as the culture supernatant of 231/si and found that the number of THP1-derived macrophages transmigrated through the membrane into the wells supplemented with the MDA-MB-231 culture supernatant was significantly higher than that with the culture supernatant of 231/si (Figure 2G). Compared with 231-EVs, EVs derived from *SIPA1*-knockdown MDA-MB-231 cells failed to promote macrophage infiltration (Figure 2H). Next, we established an MCF7/*SIPA1* cell line with the stable high-expressing SIPA1 (Figure S3A). On the contrary, macrophage migration was significantly enhanced by the treatment with MCF7/*SIPA1*-derived EVs (MCF7/*SIPA1*-EVs), whereas the treatment with MCF7-EVs at the same concentration failed to promote macrophage migration (Figure 2I). These results strongly suggested that the migration of THP1-derived macrophages was enhanced by EVs derived from breast cancer cells expressing a high level of SIPA1. The physicochemical properties of 231/si-EVs were characterised by performing Western blotting, NTA, TEM, and confocal analysis (Figure S3B,E). Next, we established a BT549/sh-*SIPA1* cell line with the stable knockdown of *SIPA1* (Figure S3F). Compared with BT549-derived EVs, EVs derived from BT549/sh-*SIPA1* cells failed to promote macrophage infiltration (Figure S3G). In addition to the THP1-derived macrophages, the migration of RAW264.7, a mouse macrophage cell line, was also significantly enhanced by treatment with 231-EVs, whereas 231/si-EVs failed to induce the migration of RAW264.7 (Figure S3H). These results indicated that EVs derived from SIPA1-expressing breast cancer cells could facilitate the migration of macrophages.

### 3.3. SIPA1-Expressing Breast Cancer Cell-Derived EVs Promote Macrophage Infiltration In Vivo and Tumour Metastasis

**Figure 3:**
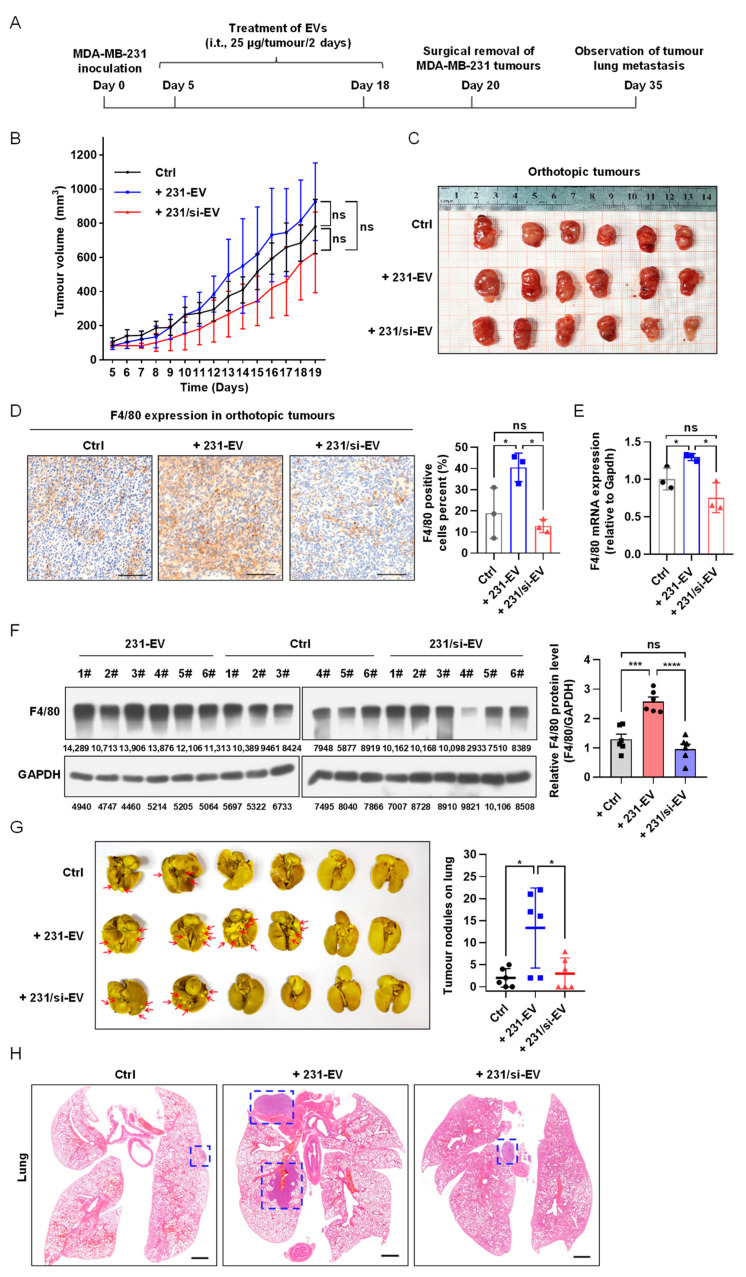
EVs derived from breast cancer cells expressing a high level of SIPA1 increase the infiltration of macrophages into tumour tissues and promote tumour metastasis. (**A**) Schematic diagram of the schedule for an MDA-MB-231 orthotopic nude mouse model (i.t, intratumoural injection). (**B**) Tumour growth curves of MDA-MB-231 cell orthotopic mice treated with EVs from day 5 to 19. Tumour growth is presented as the mean of tumour volume ± SEM (n = 6 mice/group). Data of tumour volumes on day 19 were analysed by one-way ANOVA. Ctrl, control group; ns, not significant. (**C**) Images of the tumours excised from orthotopic mice. (**D**) Immunohistochemical imaging (**left** panel) and quantitative analysis (**right** panel, n = 3) of F4/80-positive macrophages in orthotopic tumour after treatment with different EVs. Scale bar: 100 μm; ns, not significant; * *p* < 0.05. (**E**) Detection of F4/80 mRNA level in xenograft tumours treated with EVs by qRT-PCR (n = 3); ns, not significant; * *p* < 0.05. (**F**) The expression level (**left** panel) and quantitative results (**right** panel) of F4/80 protein in orthotopic tumours. Data are shown as the means ± SEM (n = 6 mice/group); ns, not significant; *** *p* < 0.001; **** *p* < 0.0001. (**G**) Representative images (**left** panel) and quantitative analysis (**right** panel) of lung tissue with metastatic tumour nodules on the surface (the red arrows indicated the tumour lesions). Data are presented as means ± SEM (n = 6 mice/group); * *p* < 0.05. (**H**) Representative images of the H&E-stained lung tissues. The blue-dash line box indicates the area of the metastatic tumour. Scale bar: 1 mm.

To examine the effect of EVs derived from SIPA1-expressing breast cancer cells on the infiltration of macrophages in vivo, we employed an animal model based on MDA-MB-231 orthotopic mice and treated mice with 231-EVs (n = 6), 231/si-EVs (n = 6), or PBS (n = 6) as control. The orthotopic experimental workflow is presented in Figure 3A. There were no statistically significant differences in the tumour volume (on day 19) and body weight among the three groups of orthotopic mice model (Figure 3B,C and Figure S4A). However, based on the results of the immunohistochemical analysis, it is worthy of note that a significant number of F4/80-positive cells infiltrated the tumour milieu (Figure 3D), and the mRNA and protein levels of F4/80 in orthotopic tumours treated with 231-EVs were higher than those with 231/si-EVs or PBS (Figure 3E,F). Furthermore, immunohistochemical staining showed that the expression levels of CD86 and CD206 were also higher in orthotopic tumours treated with 231-EVs than those with 231/si-EVs or PBS (Figure S4B). The results demonstrated that 231-EVs promoted the infiltration of mouse macrophages into the tumour microenvironment, whereas 231/si-EVs could not influence macrophage infiltration. It has been reported that the accumulation of TAMs contributes to tumour metastasis [54,55]; hence, we examined the effect of EVs derived from SIPA1-expressing breast cancer cells on the metastasis of orthotopic tumours. After the mice were sacrificed, lung tissues were carefully excised for the observation of metastatic lesions. The average number of metastatic tumour nodules on the surface of lung tissues in mice treated with 231-EVs was around 13, which was significantly higher than that in mice treated with 231/si-EVs or PBS (Figure 3G). Representative microscopic images of the H&E-stained lungs are shown in Figure 3H. It indicated that EVs derived from breast cancer cells expressing a high level of SIPA1 could recruit macrophages into the tumour milieu and promoted tumour metastasis.

### 3.4. SIPA1 Upregulates MYH9 Expression in Breast Cancer Cells and Facilitates the Accumulation of Myosin-9 in EVs

**Figure 4:**
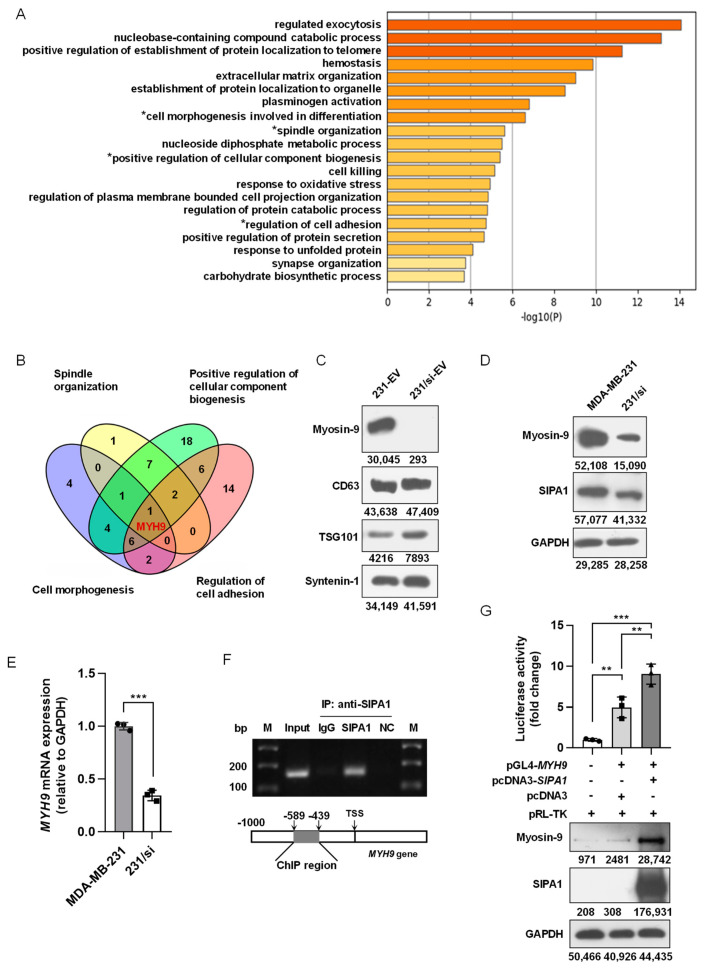
SIPA1 upregulates *MYH9* expression in breast cancer cells and facilitates the accumulation of myosin-9 in EVs. (**A**) Gene ontology (GO) analysis of 158 protein candidates exclusively in 231-EVs. The top twenty terms in the GO analysis are depicted as a bar graph. (**B**) Venn diagram of the four GO terms related to cell motility. (**C**) Detection of myosin-9, CD63, TSG101, and syntenin-1 by Western blotting in EVs. (**D**) Detection of myosin-9 and SIPA1 by Western blotting in MDA-MB-231 and 231/si cells. (**E**) Detection of *MYH9* mRNA levels in MDA-MB-231 and 231/si cells by qRT-PCR (n = 3); *** *p* < 0.001. (**F**) Chromatin immunoprecipitation (ChIP) PCR analyses for the interaction between SIPA1 and the *MYH9* promoter region. NC: Negative controls. M: DNA marker ladders. (**G**) The *MYH9* promoter activity measurement by dual-luciferase reporter assay in HEK293T cells (upper panel, n = 3), and the expression detection of SIPA1 and myosin-9 by Western blotting (lower panel); ** *p* < 0.01; *** *p* < 0.001.

To identify the components of EVs that might be involved in the regulation of macrophage migration, we applied an LC-ESI MS/MS spectrometer for proteomics analysis and compared the protein profiles of 231-EVs and 231/si-EVs. As shown in Figure S5A, a total of 307 proteins and 188 proteins were identified in the 231-EVs and 231/si-EVs, respectively. Next, we selected 158 proteins preferentially or selectively expressed in 231-EVs and analysed the protein candidates by conducting a GO enrichment analysis of the biological process. Among the top 20 GO biological process terms (Figure 4A), four terms were related to cell motility: “Spindle Organisation”, “Positive Regulation of Cellular Component Biogenesis”, “Cell Morphogenesis Involved in Differentiation”, and “Regulation of Cell Adhesion” (marked with asterisks in Figure 4A). As shown in Figure 4B, myosin-9 encoded by *MYH9* was the only candidate present in all four terms. Additionally, myosin-9 was the only candidate that matched over 20 unique peptides among the 158 protein candidates identified by MS, strongly suggesting that myosin-9 in the 231-EVs was involved in the regulation of macrophage migration (Table S3).

Myosin-9 promotes gastric cancer cell invasion and metastasis [56,57,58] and facilitates cell growth and metastasis in colorectal and pancreatic cancers [59,60]. As shown in Figure 4C, myosin-9 was highly expressed in 231-EVs, whereas the expression of myosin-9 was marginal in 231/si-EVs. When it comes to the expression levels of myosin-9 in breast cancer cells, MDA-MB-231 cells showed a higher expression of myosin-9 than 231/si cells (Figure 4D), and the mRNA expression of *MYH9* in 231/si cells was significantly lower than that in MDA-MB-231 cells (Figure 4E). We also confirmed that the overexpression of SIPA1 upregulated myosin-9 expression in MCF7 cells (Figure S5B). These results suggest that a high expression of SIPA1 leads to the upregulation of *MYH9* transcription in breast cancer cells.

Our previous study reported that SIPA1 was located in the nucleus of MDA-MB-231 cells and induced the promoter activity of integrin β1, which led to the aggravation of metastasis [25]. The ChIP-PCR assay revealed that SIPA1 interacted with the promoter region of the *MYH9* gene in MDA-MB-231 cells (Figure 4F). Luciferase reporter gene assay and Western blotting analysis further confirmed that SIPA1 could activate *MYH9* transcription and enhance endogenous *MYH9* expression in HEK293T cells (Figure 4G). These results strongly suggested that SIPA1 could bind to the *MYH9* promoter region and induced its transcriptional activity in breast cancer cells, resulting in an increase in the myosin-9 level in EVs.

### 3.5. Myosin-9 in EVs Derived from Breast Cancer Cells Is a Key Factor for Macrophage Migration

**Figure 5:**
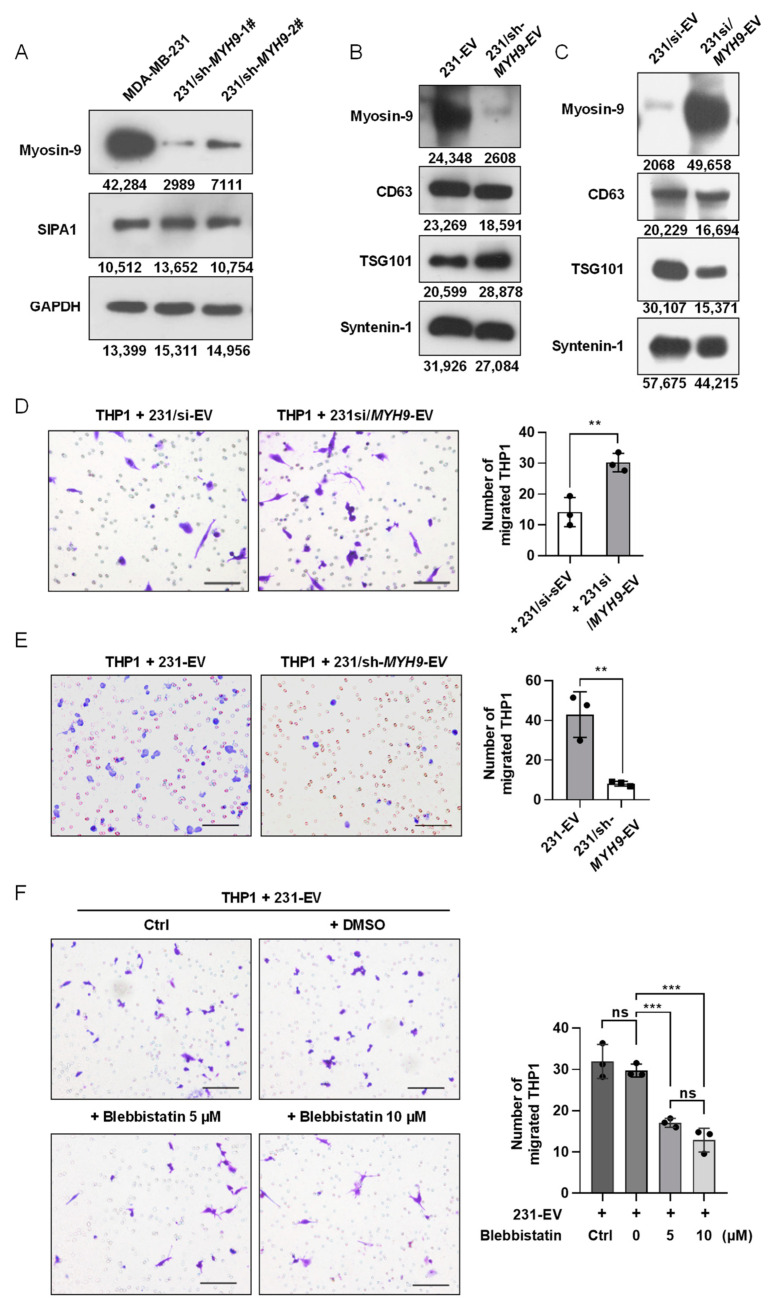
Myosin-9 in EVs derived from breast cancer cells is a key factor for macrophage migration. (**A**) Detection of myosin-9 and SIPA1 by Western blotting in MDA-MB-231 and 231/sh-*MYH9* cells. (**B**) Detection of myosin-9, TSG101, CD63, and syntenin-1 by Western blotting in EVs. (**C**) Detection of myosin-9, CD63, TSG101, and syntenin-1 by Western blotting in EVs derived from 231/si cells and *MYH9*-overexpressed 231/si cells (231si/*MYH9*-EVs). (**D**) Representative images (**left** panel) and quantitative analysis (**right** panel, n = 3) of migrated macrophages treated with 231/si-EVs (200 μg/mL) or 231si/*MYH9*-EVs (200 μg/mL) by transwell assay. Scale bar: 100 μm; ** *p* < 0.01. (**E**) Representative images (**left** panel) and quantitative analysis (**right** panel, n = 3) of migrated macrophages treated with 231-EVs (200 μg/mL) or EVs (200 μg/mL) derived from 231/sh-*MYH9* cells (231/sh-*MYH9*-EVs) by transwell assay. Scale bar: 100 μm; ** *p* < 0.01. (**F**) Representative images (**left** panel) and quantification (**right** panel, n = 3) of migrated macrophages treated with 231-EVs (200 μg/mL) in the presence of a serial dilution of blebbistatin by transwell assays. Scale bar: 100 μm; ns, not significant; *** *p* < 0.001.

To examine the effect of myosin-9 on the migration of macrophages, we established a stable *MYH9*-knockdown MDA-MB-231 cell line (231/sh-*MYH9*) as shown in Figure 5A. Notably, SIPA1 expression was not altered when *MYH9* was suppressed in MDA-MB-231 cells, indicating that SIPA1 regulated the expression of *MYH9*, but not vice versa.

Therefore, we tested whether modulating the expression level of *MYH9* in breast cancer cells would affect the accumulation of myosin-9 in EVs. When the expression of myosin-9 was examined by Western blotting analysis, no detectable band corresponding to myosin-9 was observed in EVs derived from 231/sh-*MYH9* cells (231/sh-*MYH9*-EVs) (Figure 5B). We also transfected pCMV7.1-*MYH9* plasmids into 231/si cells and found that the expression levels of myosin-9 were upregulated in both 231/si cells and EVs (Figure S6A and Figure 5C). These results indicated that the accumulation of myosin-9 in EVs was regulated by modulating the expression level of *MYH9* in cells.

To verify the effect of myosin-9 in EVs on the migration of macrophages, THP1-derived macrophages were treated with EVs derived from *MYH9*-overexpressing 231/si cells (231si/*MYH9*-EVs), and then the number of macrophages that migrated across the transwell membrane was significantly higher than that with 231/si-EVs (Figure 5D). Next, we examined the effect of 231/sh-*MYH9*-EVs on the migration of macrophages. When THP1-derived macrophages were treated with 231/sh-*MYH*9-EVs, the number of macrophages that migrated across the transwell membrane was significantly lower than those from parental MDA-MB-231 cells (Figure 5E). Therefore, the knockdown of *MYH9* in breast cancer cells reduced the expression of myosin-9 in both cells and EVs, leading to the suppression of macrophage migration. Furthermore, we employed blebbistatin, a specific myosin-9 inhibitor, in the transwell assay system, to validate the effect of myosin-9 on macrophage migration. When THP1-derived macrophages were treated with 10 μmol/L blebbistatin, essentially no cytotoxic effect was observed (Figure S6B). Next, we examined the effect of blebbistatin on the migration of macrophages treated with 231-EVs. The addition of blebbistatin significantly inhibited the migration of macrophages (Figure 5F). These results show that up-regulation of *MYH9* expression in breast cancer cells can promote the accumulation of myosin-9 in both cells and EVs, and myosin-9-enriched EVs can enhance the migration of macrophages.

### 3.6. MYH9 Expression Positively Correlates with Poor Prognosis in Triple-Negative Breast Cancer

**Figure 6:**
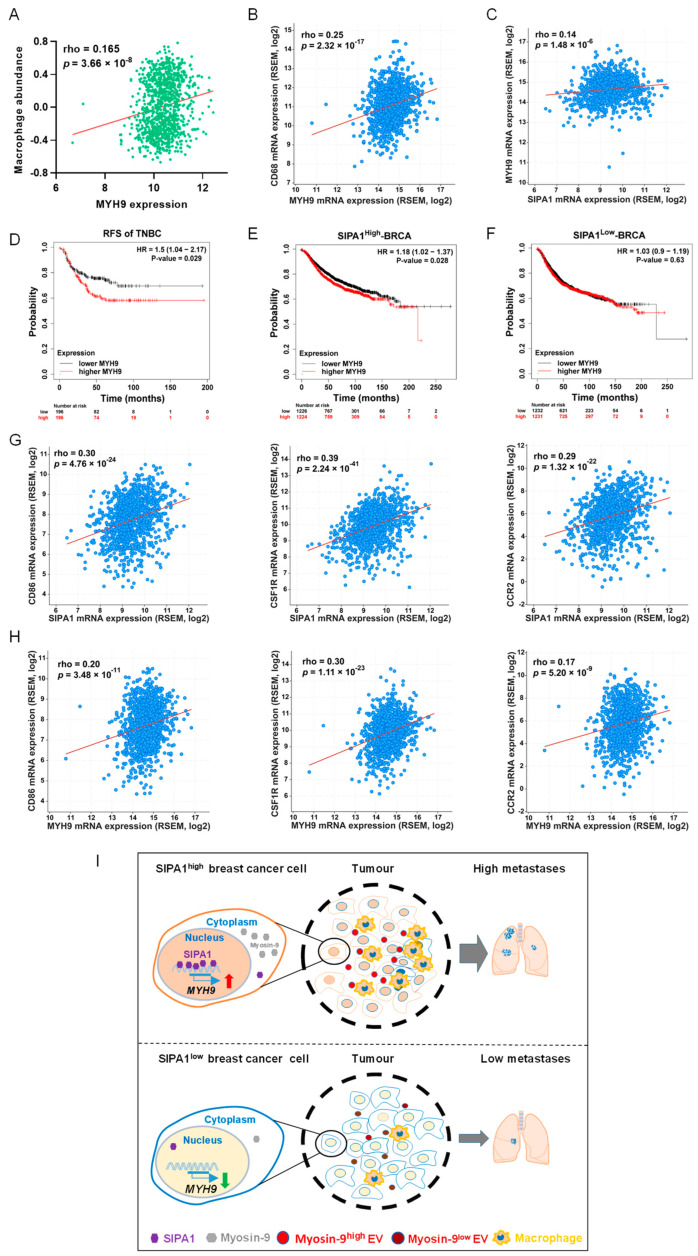
*MYH9* is a poor prognosis predictor of triple-negative breast cancer. (**A**) The correlation between *SIPA1* expression and macrophage abundance based on the TISIDB database (n = 1100). (**B**) Co-expression analysis of *MYH9* and *CD68* in breast cancer via cBioPortal platform (n = 1100). (**C**) Co-expression analysis of *SIPA1* and *MYH9* in breast cancer via cBioPortal platform (n = 1100). (**D**) Kaplan–Meier survival curves based on *MYH9* expression for relapse-free survival (RFS) in triple-negative breast cancer (TNBC). (**E**,**F**) Kaplan–Meier survival curves based on *MYH9* expression for relapse-free survival (RFS) in SIPA1^high^-BRCA (**E**) and SIPA1^low^-BRCA (**F**). (**G**,**H**) Co-expression analysis of tumour-associated macrophages (*CD86*, *CSF1R*, and *CCR2*) correlated with *SIPA1* (**G**) and *MYH9* (**H**) in breast cancer via cBioPortal platform, respectively (n = 1100). (**I**) Schematic diagram illustrating SIPA1-induced myosin-9 expression and subsequent infiltration of macrophages and tumour metastasis.

By analysing the transcriptome data of the TCGA-BRCA database, we found that the mRNA expression level of *MYH9* positively correlated with macrophage abundance and *CD68*, respectively (Figure 6A,B). In addition, we also found a positive correlation between mRNA levels of *MYH9* and *SIPA1* genes in breast cancer (Figure 6C). Our analyses revealed that the relapse-free survival (RFS) of BRCA, HER2 positive, luminal A, and luminal B did not significantly correlate with *MYH9* expression except for triple-negative breast cancer (TNBC) (*p* = 0.029) (Figure S7A–D and Figure 6D).

Moreover, the RFS of SIPA1^high^ breast cancer patients with a high *MYH9* expression was significantly lower than in patients with low *MYH9* expression, while the RFS of BRCA patients with SIPA1^low^ expression was not significant (Figure 6E,F). Furthermore, we found that, in breast cancer, the expression of three typical markers of TAMs (*CD86*, *CSF1R*, and *CCR2*) were positively correlated with *SIPA1* and *MYH9*, respectively (Figure 6G,H). It suggests that macrophages in breast cancer may be an important factor leading to the decreased RFS of breast cancer patients with high expression of *SIPA1* and *MYH9*.

Taken together, SIPA1 induced *MYH9* transcription and up-regulated its expression at the protein level in breast cancer cells. EVs released from breast cancer cells enriched with myosin-9 promoted the migration of macrophages, resulting in the infiltration of macrophages into the tumour microenvironment and the promotion of cancer cell metastasis (Figure 6I).

## 4. Discussion

SIPA1 is ectopically localised in the nucleus in some breast cancer cells and could regulate the expression of certain genes, leading to the promotion of cancer cell metastasis [20,27]. Here, we first demonstrated that macrophages infiltrated tumour tissues in breast cancer patients with a high level of SIPA1 expression. We next showed that EVs derived from highly metastatic breast cancer cells induced macrophage migration in vitro and that the level of SIPA1 expression in breast cancer cells may influence the components of EVs and the migration of macrophages. Furthermore, animal models revealed that EVs secreted by breast cancer cells with a high level of SIPA1 expression recruited more macrophages into tumour tissues and promoted metastasis of tumour cells to the lung tissues. These findings suggest that SIPA1 induced the expression of certain genes that are responsible for the migration of macrophages. These macrophages are considered TAMs and are associated with tumour growth and metastasis.

We analysed the components of EVs derived from the SIPA1-highly expressing breast cancer cells and found that myosin-9 was selectively present in the EVs. However, we could not detect SIPA1 expression at the protein level in the 231-EVs or MCF7/*SIPA1*-EVs (Figure S8A). The exosome database Exocreta (http://www.exocarta.org, accessed on 8 December 2020) indicated that myosin-9 was among the top 100 proteins in exosomes. *MYH9* encoding myosin-9 plays dual functions in cancers. Myosin-9 is involved in the determination of cell polarity and cytoskeleton rearrangement [61,62]. We found that SIPA1 up-regulated myosin-9 levels in breast cancer cells as well as in EVs by binding to the *MYH9* gene promoter. The knockdown of the *MYH9* gene in MDA-MB-231 cells reduced the expression of myosin-9 in both cells and EVs and significantly suppressed the migration of MDA-MB-231 cells (Figure S8B). We also found that blebbistatin, an *MYH9* inhibitor, suppressed the migration of macrophages induced by 231-EVs. Myosin-9-containing EVs enhanced the migration of macrophages and increased the infiltration of macrophages into tumour tissues. The inhibition or down-regulation of myosin-9 reduced the metastasis of cancer cells [63] and also suppressed the infiltration of surrounding macrophages. The results indicate that blebbistatin is a potential therapeutic agent that can inhibit the recruitment of macrophages, providing a preclinical basis for the development of inhibitors of breast cancer cell metastasis.

## 5. Conclusions

In conclusion, SIPA1 in breast cancer cells promoted *MYH9* transcription and up-regulated the expression of myosin-9. Moreover, myosin-9-enriched EVs derived from breast cancer cells promoted the infiltration of macrophages into the tumour microenvironment, resulting in cancer metastasis.

## Data Availability

The data presented in this study are available on request from the corresponding author.

## Supplementary Materials

The following supporting information can be downloaded at: https://www.mdpi.com/article/10.3390/biology11040543/s1, Figure S1: SIPA1 expression levels in breast cancer cells and the expression of the macrophage markers in xenograft tumours, Figure S2: Extracellular vesicles (EVs) isolation procedures and the migration of THP1-derived macrophages after treatment with the 231-EVs, Figure S3: Identification of EV and the migration of macrophages after the treatment with the different EVs, Figure S4: Body weight curve of mice and the expression of macrophage markers in orthotopic tumours, Figure S5: The expression level of myosin-9 in parental and SIPA1-overexpression MCF7 cells, Figure S6: Overexpression of *MYH9* in 231/si cells and the cell viability of THP1-derived macrophages after treated with different concentrations of blebbistatin, Figure S7: Kaplan–Meier survival curves of relapse-free survival (RFS), Figure S8: The expression detection of SIPA1 in EVs and the migration ability of MDA-MB-231 cells after knocking down *MYH9*, Table S1: The information of primers used in this study, Table S2: Antibodies list used in this study, Table S3: Top 10 unique proteins in the 231-EVs.
