# Supplementary material for: Extracellular Vesicles Derived from SIPA1high Breast Cancer Cells Enhance Macrophage Infiltration and Cancer Metastasis through Myosin-9
Source: Biology (Basel). 2022 Mar 31;11(4):543. doi: 10.3390/biology11040543 (PMC9032110; doi:10.3390/biology11040543)
Supplement: Supplementary file 1 [file biology-11-00543-s001.zip › biology-1623509-supplementary revised.pdf]

Supplementary information

Supplementary figures

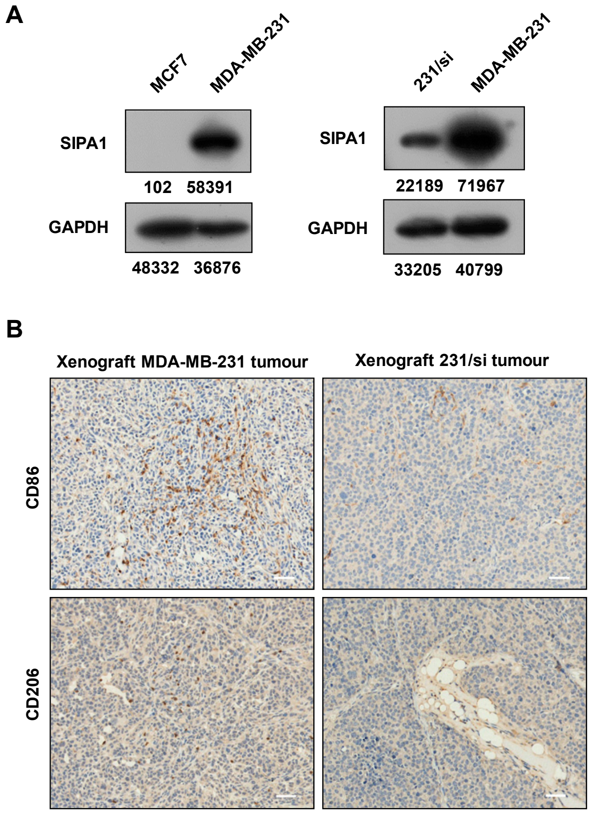

Figure S1: SIPA1 expression levels in breast cancer cells and the expression of the macrophage markers in xenograft tumours. **(A)** SIPA1 expression in MCF7, MDA-MB-231, and SIPA1 knockdown MDA-MB-231 (231/si) by western blotting analysis. GAPDH was used as an internal reference control. **(B)** Immunohistochemical analysis of the macrophage marker CD86 and CD206 in MDA-MB-231 or 231/si cell xenograft tumours. Scale bar: 50 μm.

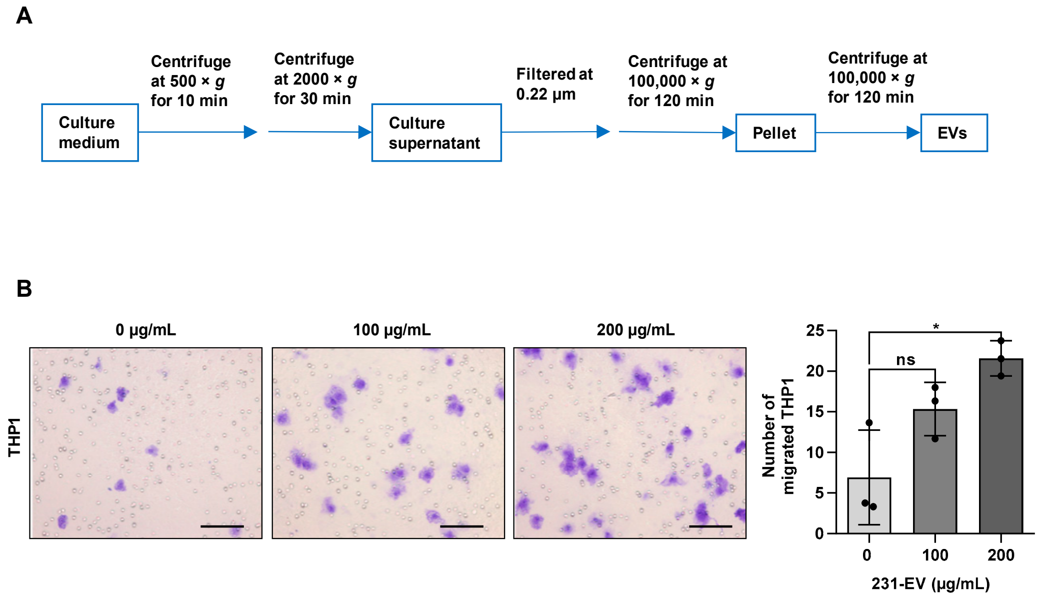

Figure S2: Extracellular vesicles (EVs) isolation procedures and the migration of THP1-derived macrophages after the treatment with the 231-EVs. **(A)** Schematic diagram illustrating the isolation of extracellular vesicles. **(B)** Representative images (left panel) and quantitative analysis (right panel, n = 3) of the infiltration of THP1-derived macrophages after the treatment with different concentrations of 231-EVs. Scale bar, 100 μm.

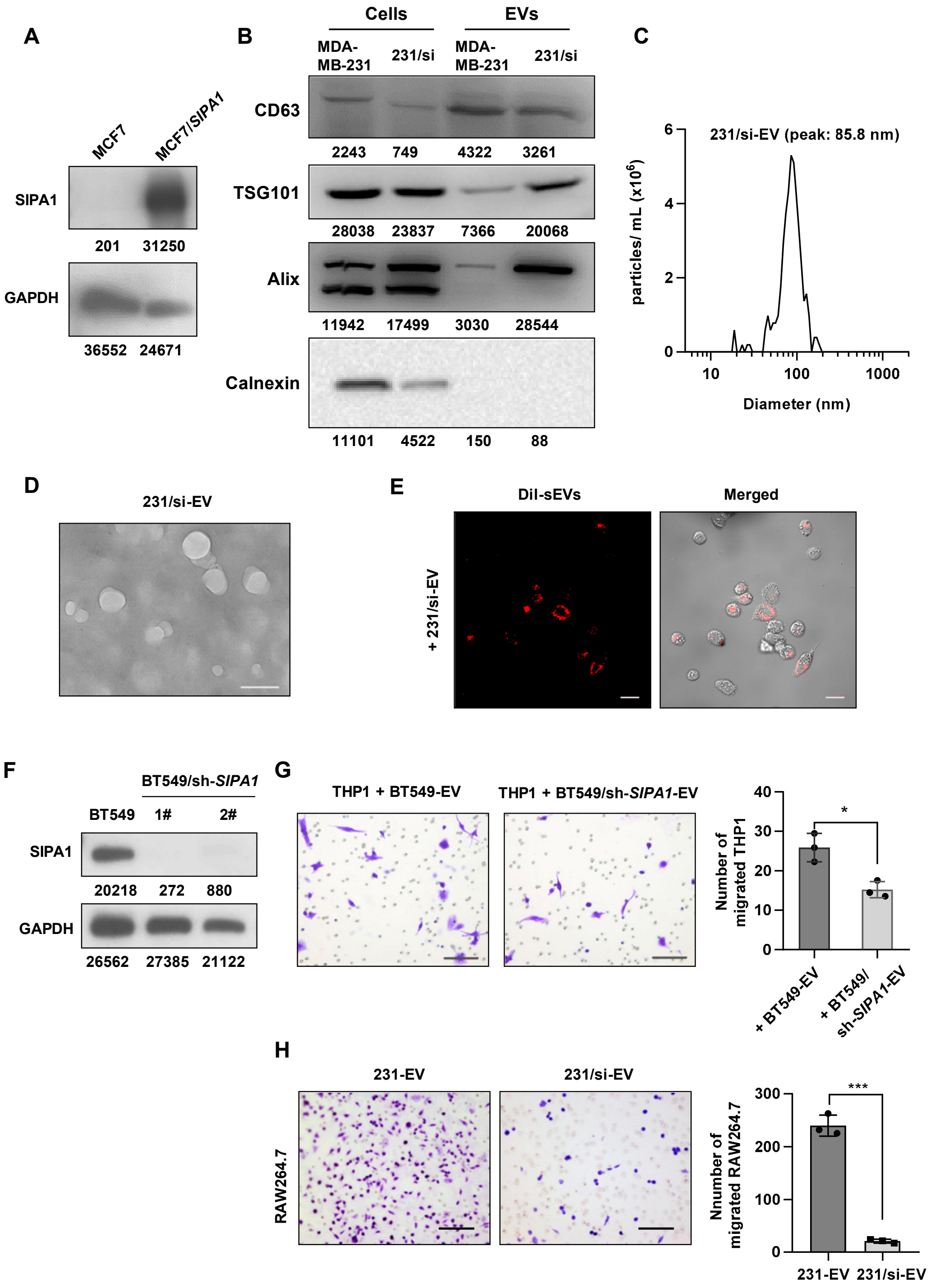

Figure S3: Identification of EV and the migration of macrophages after the treatment with the different EVs. **(A)** SIPA1 expression in MCF7 and SIPA1-overexpressed MCF7 (MCF7/*SIPA1*) cells by western blotting analysis. GAPDH was used as an internal reference control. **(B)** Western blotting analysis the expressions of CD63, TSG101, Alix, and calnexin in whole cell and EV lysates. **(C)** The diameter measurement of 231/si-EVs by nanoparticle tracking analysis. **(D)** TEM images of 231-EVs and EVs derived from 231/si cells (231/si-EVs). Scale

bar, 200 nm. **(E)** Confocal microscopic imaging of the internalization of EVs into THP1-derived macrophages. The fluorescence of Dil-stained EVs is shown in red. Scale bar, 20 μm. **(F)** Detection of SIPA1 by western blotting in BT549 and BT549/sh-*SIPA1* cells. **(G)** Representative images (left panel) and quantitative analysis (right panel, n = 3) of migrated macrophages treated with EVs (200 μg/mL) by transwell assay. Scale bar: 100 μm. **(H)** Representative images (left panel) and quantitative analysis (right panel, n = 3) of migrated RAW264.7 cells (mouse macrophages) after the treatment with 231-EVs (200 μg/mL) or 231/si-EVs (200 μg/mL). Scale bar, 100 μm.

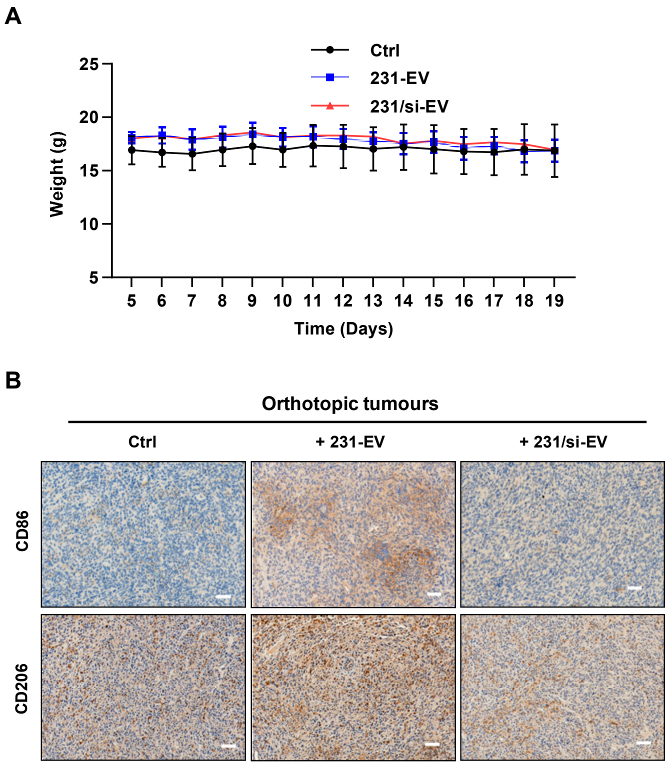

Figure S4: Body weight curve of mice and the expression of macrophage markers in orthotopic tumours. **(A)** Time courses of mice body weights for three mice groups. Data are shown as the means ± SEM (n = 6 mice/group). **(B)** Immunohistochemical imaging of CD86 and CD206 in orthotopic tumour after treatment with different EVs. Scale bar: 50 μm.

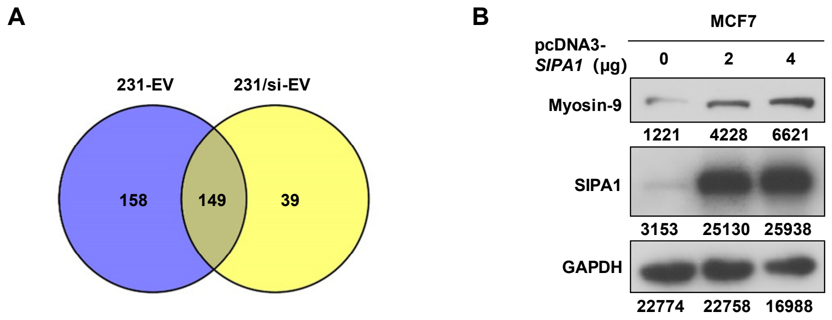

Figure S5: The expression level of myosin-9 in parental and SIPA1-overexpression MCF7 cells. **(A)** Venn diagram showing differentially expressed proteins in the 231-EVs and 231/si-EVs (n = 2). **(B)** Detection of SIPA1 and myosin-9 by western blotting in the MCF7 cells transfected with pcDNA3-SIPA1 plasmid.

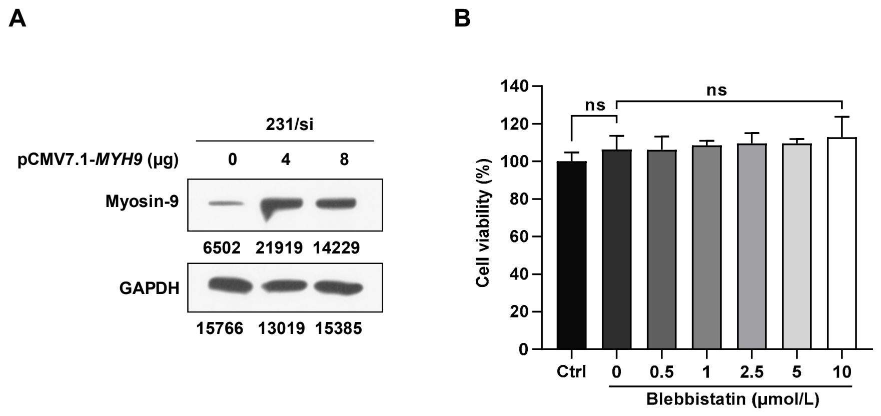

Figure S6: Overexpression of *MYH9* in 231/si cells and the cell viability of THP1-derived macrophages after treated with different concentrations of blebbistatin. **(A)** Western blotting analysis of myosin-9 in 231/si and *MYH9* overexpression 231/si cells. GAPDH was used as an internal reference control. **(B)** THP1-derived macrophages were treated with 0, 0.5, 1, 2.5, 5 or 10 μmol/L blebbistatin (DMSO = 0.1%) for 24 h. Control group (Ctrl) has no DMSO added. Cell viability was evaluated using the CCK8 Cell Viability Assay. Data are shown as mean ± SD of five independent experiments; ns, not significant (P>0.05).

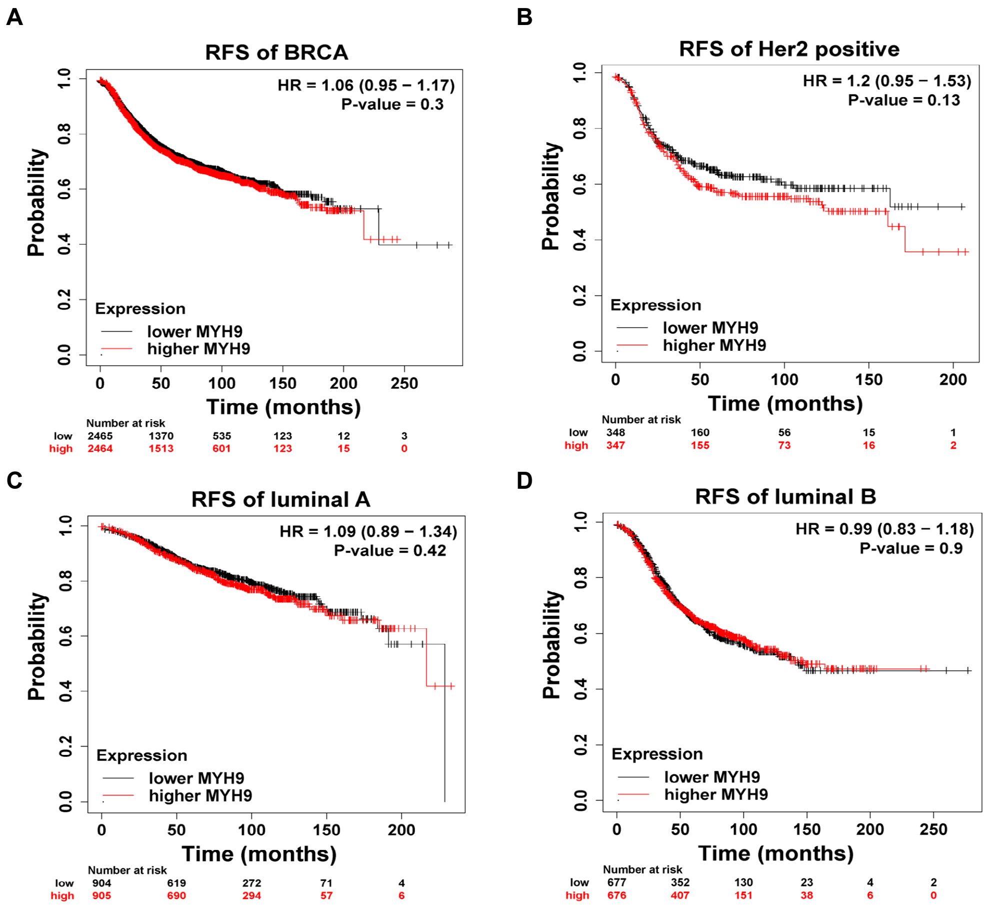

Figure S7: Kaplan-Meier survival curves of relapse-free survival (RFS). **(A-D)** Kaplan-Meier survival curves based on *MYH9* expression for relapse-free survival (RFS) in Breast invasive carcinoma (BRCA) **(A)**, HER2 positive **(B)**, luminal A **(C)**, and luminal B **(D)**.

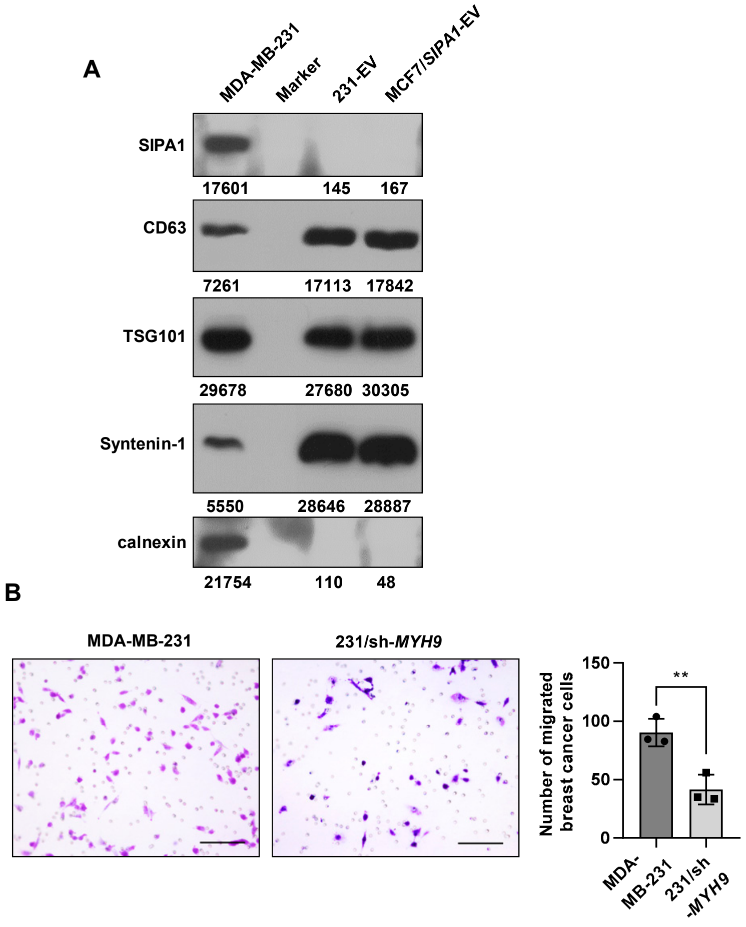

Figure S8: The expression detection of SIPA1 in EVs and the migration ability of MDA-MB-231 cells after knocking down *MYH9*. **(A)** SIPA1 protein expression in MDA-MB-231 cell, 231-EVs, and MCF7/*SIPA1*-EVs by western blotting analysis. CD63, TSG101 and syntenin-1 were included as markers of EVs. The expression of calnexin was used as a negative EV marker. **(B)** Representative images (left panel) and quantitative analysis (right panel, n = 3) of migrated MDA-MB-231 and 231/sh-*MYH9* cells by transwell assay. Scale bar: 100 μm.

Table S1. The information of primers used in this study

Gene Name	Forward Primer and Reverse Primer
Human-*MYH9*	5′-TGAGGCAGAAGCACTCACAG-3′
	5′-CACTTTCTTGCGCTTGTGCT-3′
Human-*GAPDH*	5′-GTCTCCTCTGACTTCAACAGCG-3′
	5′-ACCACCCTGTTGCTGTAGCCAA-3′
Mouse-F4/80	5′-TCTGGGGAGCTTACGATGGA-3′
	5′-GAATCCCGCAATGATGGCAC-3′
Mouse-Gapdh	5′-TGTGAACGGATTTGGCCGTA-3′
	5′-ACTGTGCCGTTGAATTTGCC-3′
MYH9-ChIP	5′-TTGAATTGCATCTGTCTTTGAGACTC-3′
	5′-ACTCCAGCCTGGGCAACAGAAAAAC-3′
MYH9-promoter	5′-CGGGGTACCTATTTTATATCTTCATTTTGCAAATGAGTT-3′
	5′-ATAAAAGCTTGTTGAGGCACGTGAGCTCTGC-3′
MYH9-shRNA-1#	5′-CCGGGACAGCAATCTGTACCGCATTCTCGAGAATGCGGTACAGATTGCTGTCTTTTTG-3′
	5′-AATTCAAAAAGACAGCAATCTGTACCGCATTCTCGAGAATGCGGTACAGATTGCTGTC-3′
MYH9-shRNA-2#	5′-CCGGGCCAAGCTCAAGAACAAGCATCTCGAGATGCTTGTTCTTGAGCTTGGCTTTTTG-3′
	5′-AATTCAAAAAGCCAAGCTCAAGAACAAGCATCTCGAGATGCTTGTTCTTGAGCTTGGC-3′
SIPA1-shRNA-1#	5′-CCGGCGCAAATACTTCTATGGCAAACTCGAGTTTGCCATAGAAGTATTTGCGTTTTTTG-3′
	5′-AATTCAAAAACGCAAATACTTCTATGGCAAACTCGAGTTTGCCATAGAAGTATTTGCG-3′
SIPA1-shRNA-2#	5′-CCGGCAAAGAACATCAGAACTTCTTCTCGAGAAGAAGTTCTGATGTTCTTTGTTTTTTG-3′

5′-AATTCAAAAACAAAGAACATCAGAACTTCTTCTCGAGAAGAAGTTC
TGATGTTCTTTG-3′

Table S2. Antibodies list used in this study

	Name	Source*	Cat. No.	Dilution
Primary antibodies	SIPA1	Abcam	ab189929	WB,1:1000; IF, 1:200
	CD68	Servicebio	GB14043	IF, 1:100
	CD63	Abcam	ab59479	WB,1:1000
	TSG101	Abcam	ab125011	WB,1:1000
	Alix	Abcam	ab186429	WB,1:1000
	Calnexin	CST	#2433	WB,1:1000
	F4/80	Servicebio	GB11027	IHC,1:400
	CD86	Servicebio	GB13585	IHC,1:400
	CD206	Servicebio	GB113497	IHC,1:400
	F4/80	CST	#30325	WB,1:1000
	Myosin-9	Abcam	ab138498	WB,1:1000
	Syntenin-1	Abcam	ab133267	WB,1:1000
	GAPDH	Servicebio	GB11002	WB,1:1000
Secondary antibodies	HRP-linked anti-rabbit	CST	#7074	1:10000
	HRP-linked anti-mouse	CST	#7076	1:10000
	CY3-linked goat anti-mouse	Servicebio	GB21303	1:300
	FITC-linked goat anti-rabbit	Servicebio	GB25303	1:400

*Antibodies provider: Abcom, Cambridge, UK; CST, Cell Signaling Technology Inc., Danvers, MA, USA; Servicebio, Wuhan, China. Abbreviations: WB, western blotting; IF, Immunofluorescence; IHC, Immunohistochemistry.

Table S3. Top 10 unique proteins in the 231-EVs

gene ID	Gene Symbol	Description	PepCount	UniquePepCount	CoverPercent
4627	*MYH9*	Myosin-9	29	28	0.1607
7431	*VIM*	Vimentin	14	12	0.2511
279	*AMY2A*	Amylase alpha 2a	11	5	0.1018
5054	*SERPINE1*	Serpin family e member 1	11	8	0.2065
87	*ACTN1*	Actinin alpha 1	10	10	0.1413
4000	*LMNA*	Lamin A/C	10	10	0.1687
3959	*LGALS3BP*	Galectin 3 binding protein	8	7	0.1265
3018	*H2BC3*	H2b clustered histone 3	7	6	0.4524
7094	*TLN1*	Talin 1	7	7	0.0315
8348	*H2BC17*	H2b clustered histone 17	7	6	0.4524
