# Supplementary figures and images for: Extracellular Vesicles Derived from SIPA1high Breast Cancer Cells Enhance Macrophage Infiltration and Cancer Metastasis through Myosin-9
Source: Biology (Basel). 2022 Mar 31;11(4):543. doi: 10.3390/biology11040543 (PMC9032110; doi:10.3390/biology11040543)

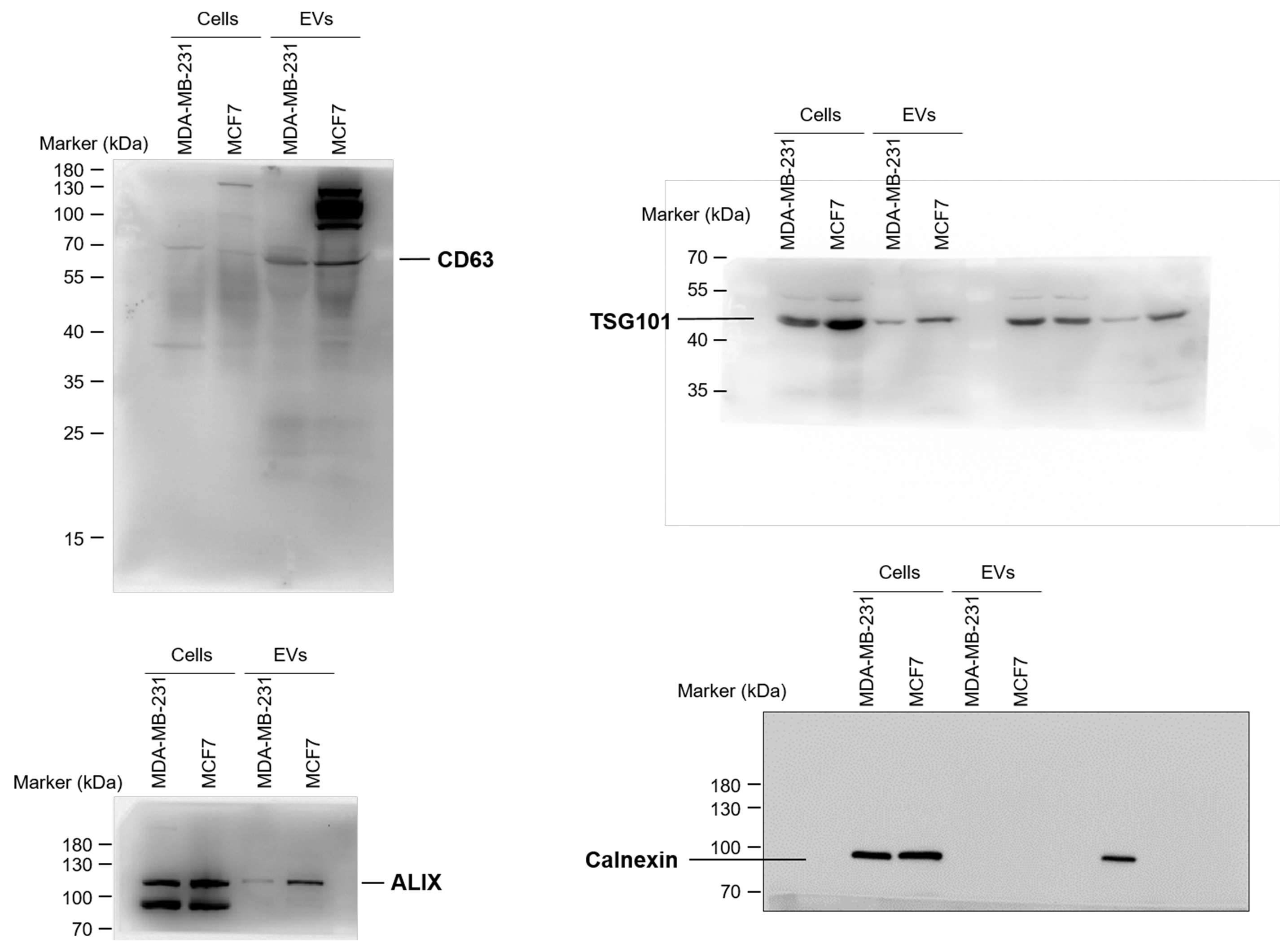

Supplement: Supplementary file 1 [file biology-11-00543-s001.zip › Original Images for western Blots and Gels/Figure 2D.tiff]

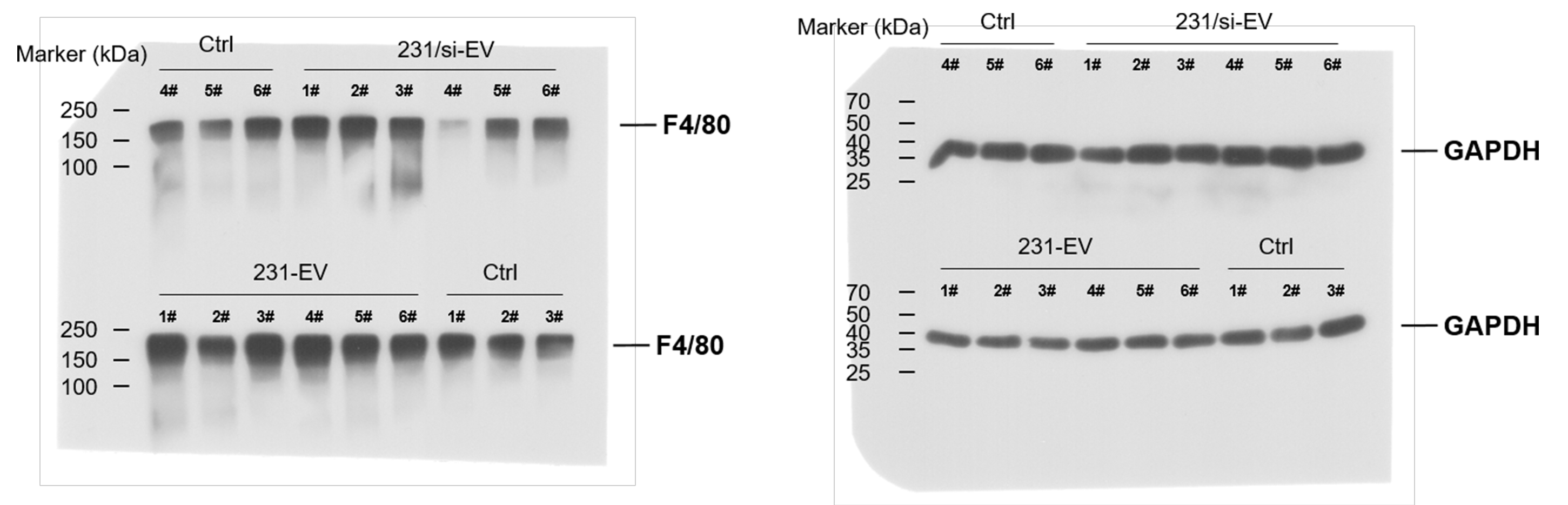

Supplement: Supplementary file 1 [file biology-11-00543-s001.zip › Original Images for western Blots and Gels/Figure 3F.tiff]

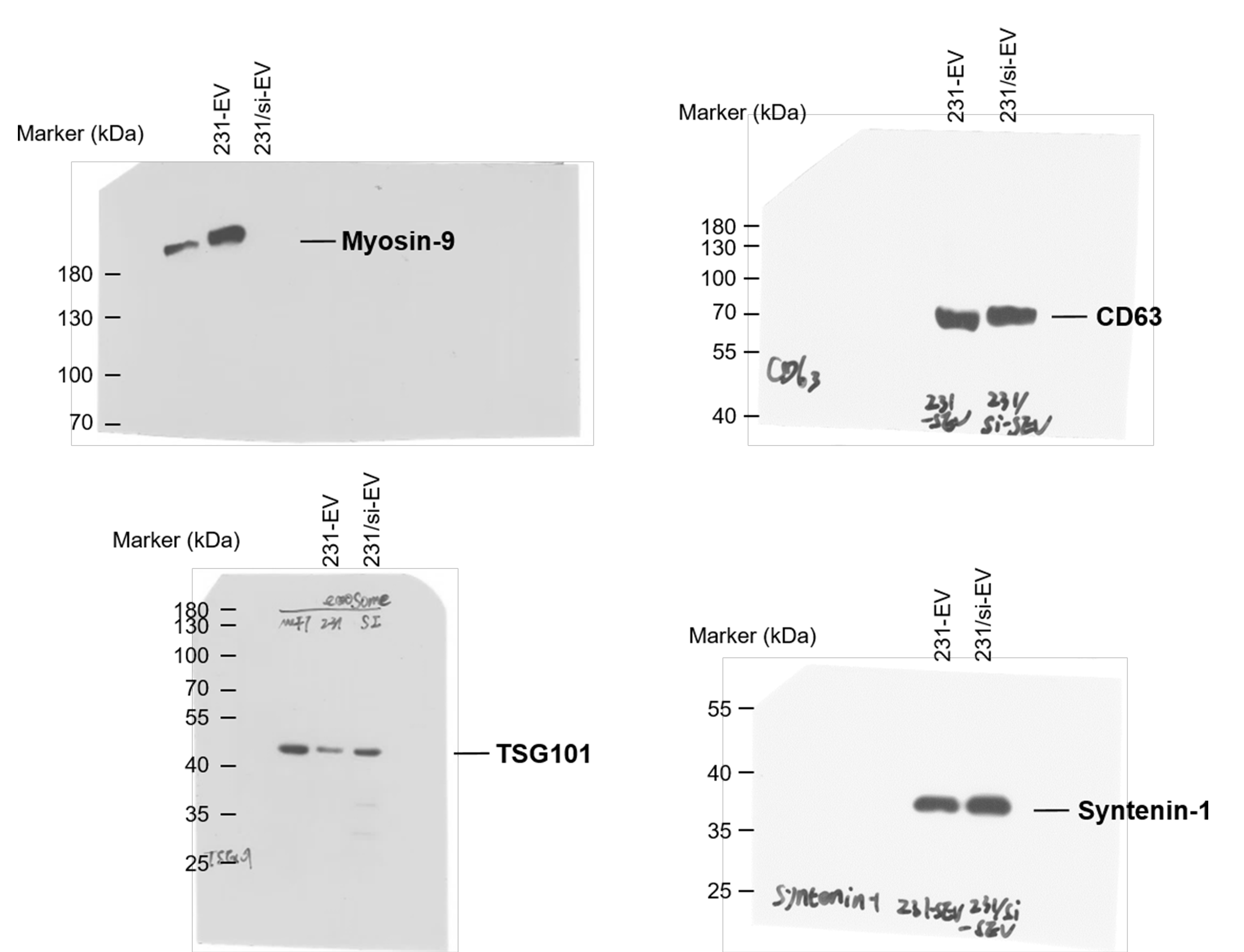

Supplement: Supplementary file 1 [file biology-11-00543-s001.zip › Original Images for western Blots and Gels/Figure 4C.tiff]

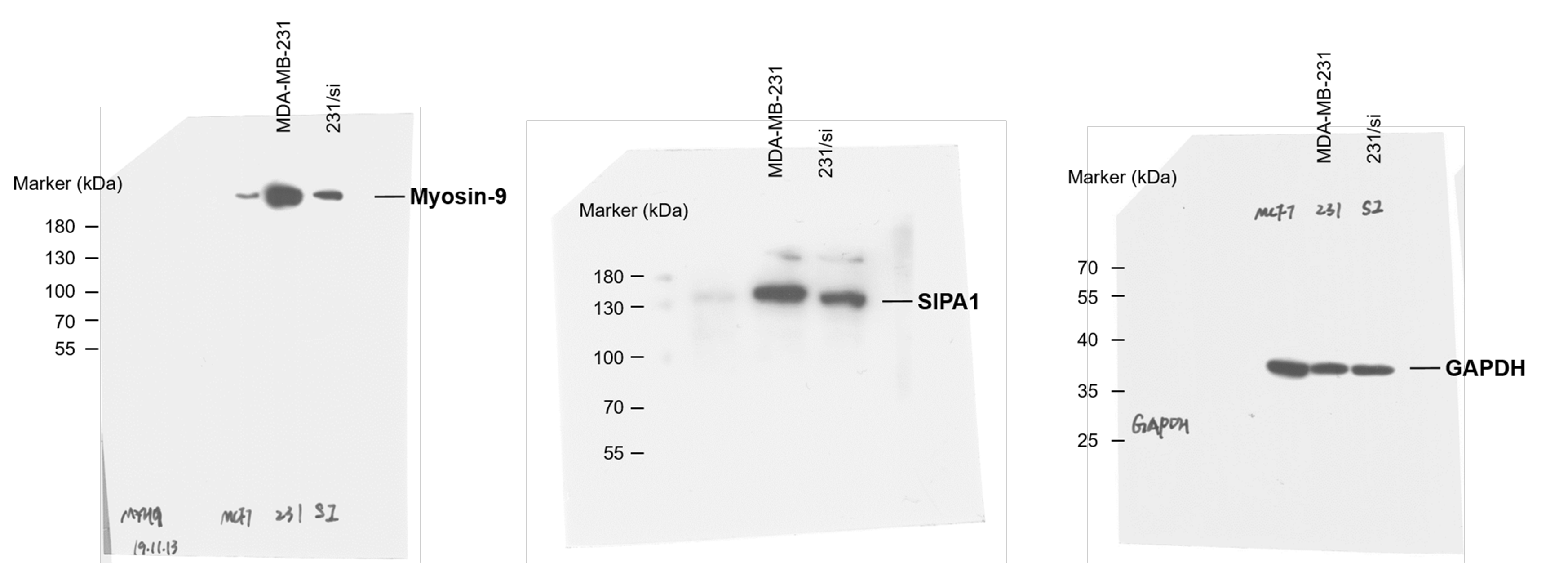

Supplement: Supplementary file 1 [file biology-11-00543-s001.zip › Original Images for western Blots and Gels/Figure 4D.tiff]

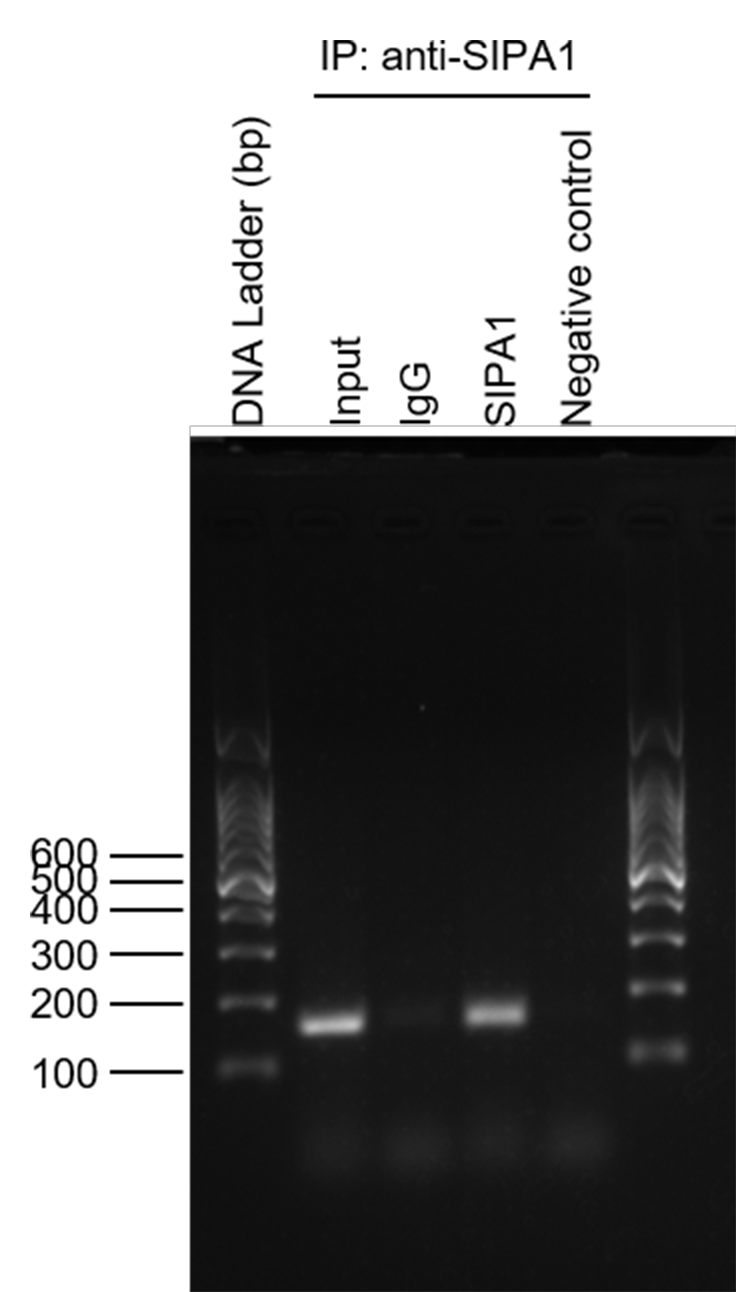

Supplement: Supplementary file 1 [file biology-11-00543-s001.zip › Original Images for western Blots and Gels/Figure 4F.tiff]

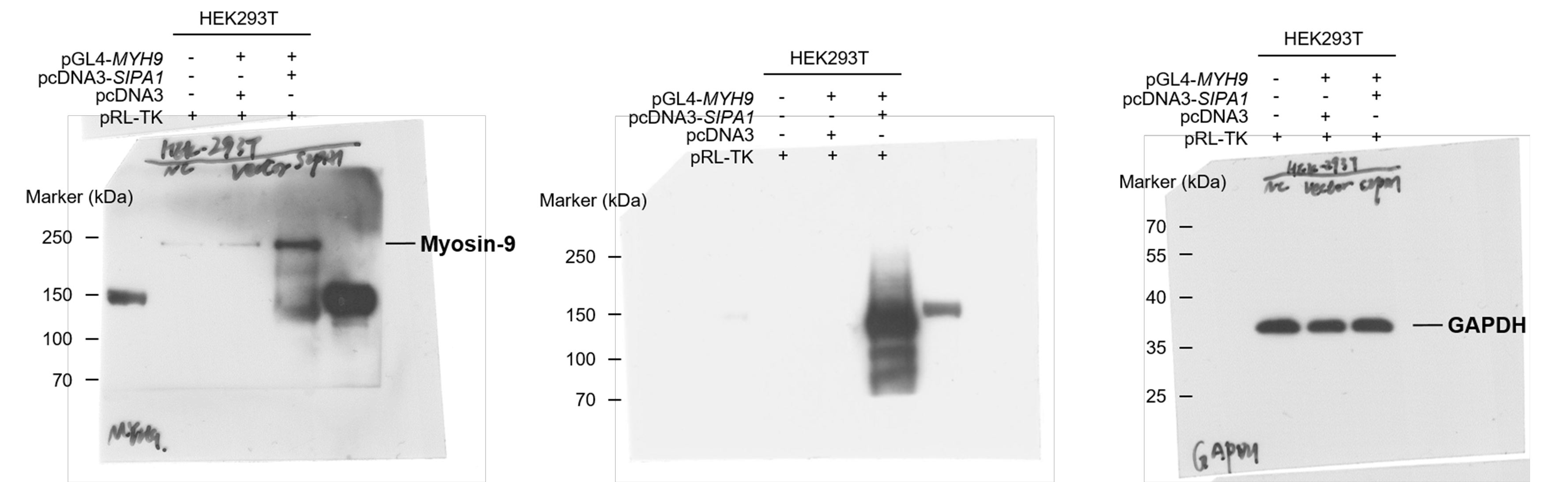

Supplement: Supplementary file 1 [file biology-11-00543-s001.zip › Original Images for western Blots and Gels/Figure 4G.tiff]

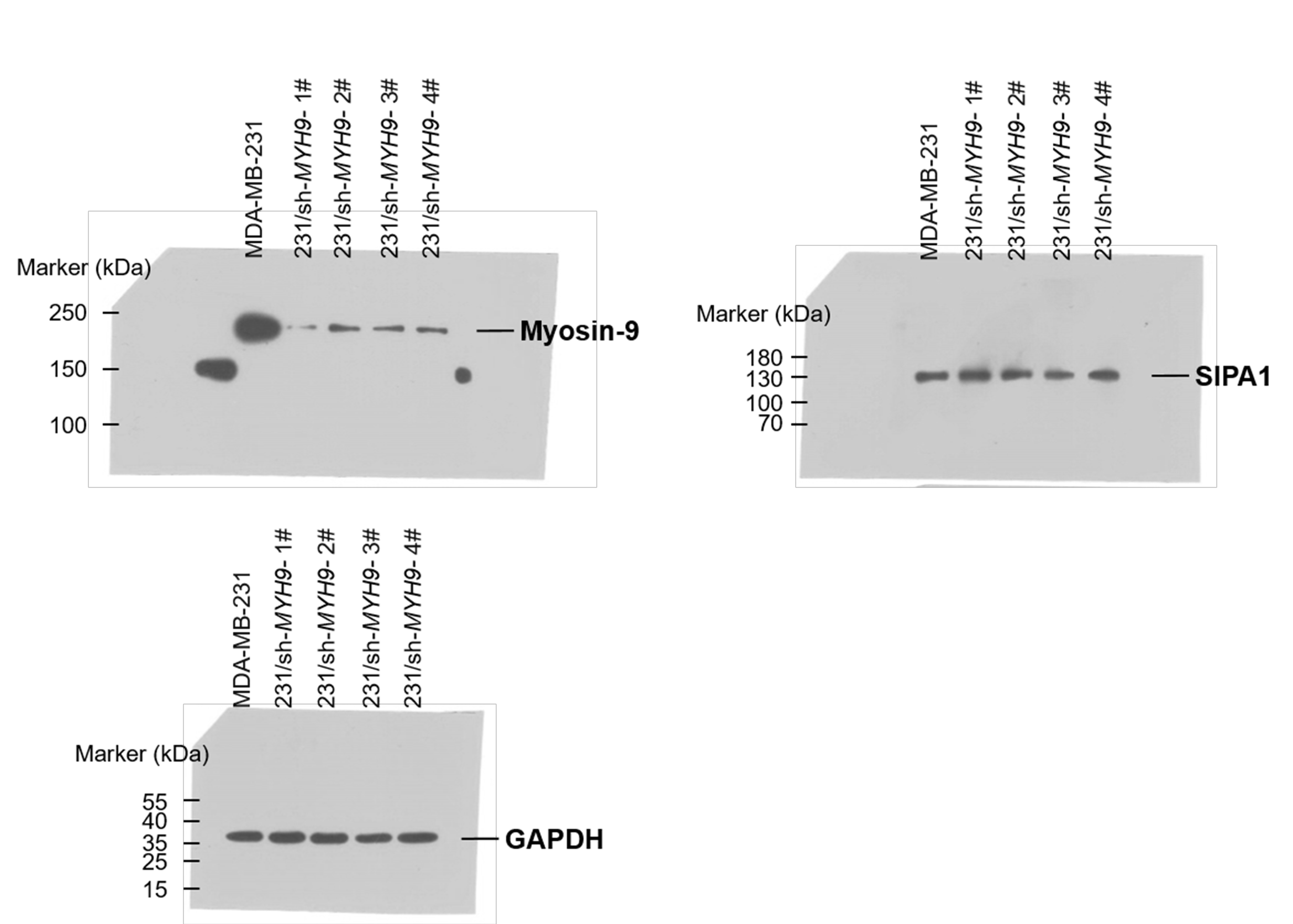

Supplement: Supplementary file 1 [file biology-11-00543-s001.zip › Original Images for western Blots and Gels/Figure 5A.tiff]

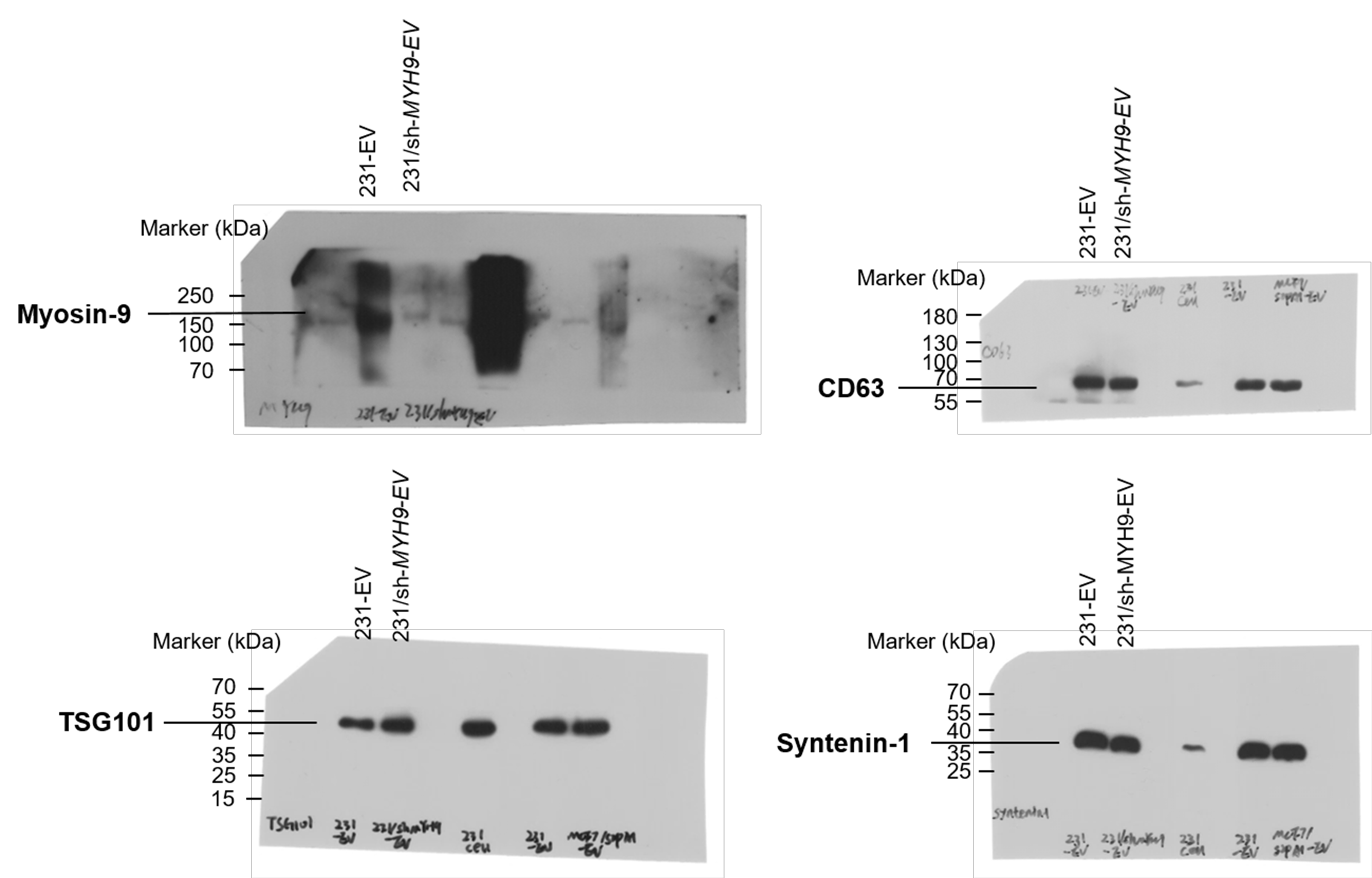

Supplement: Supplementary file 1 [file biology-11-00543-s001.zip › Original Images for western Blots and Gels/Figure 5B.tiff]

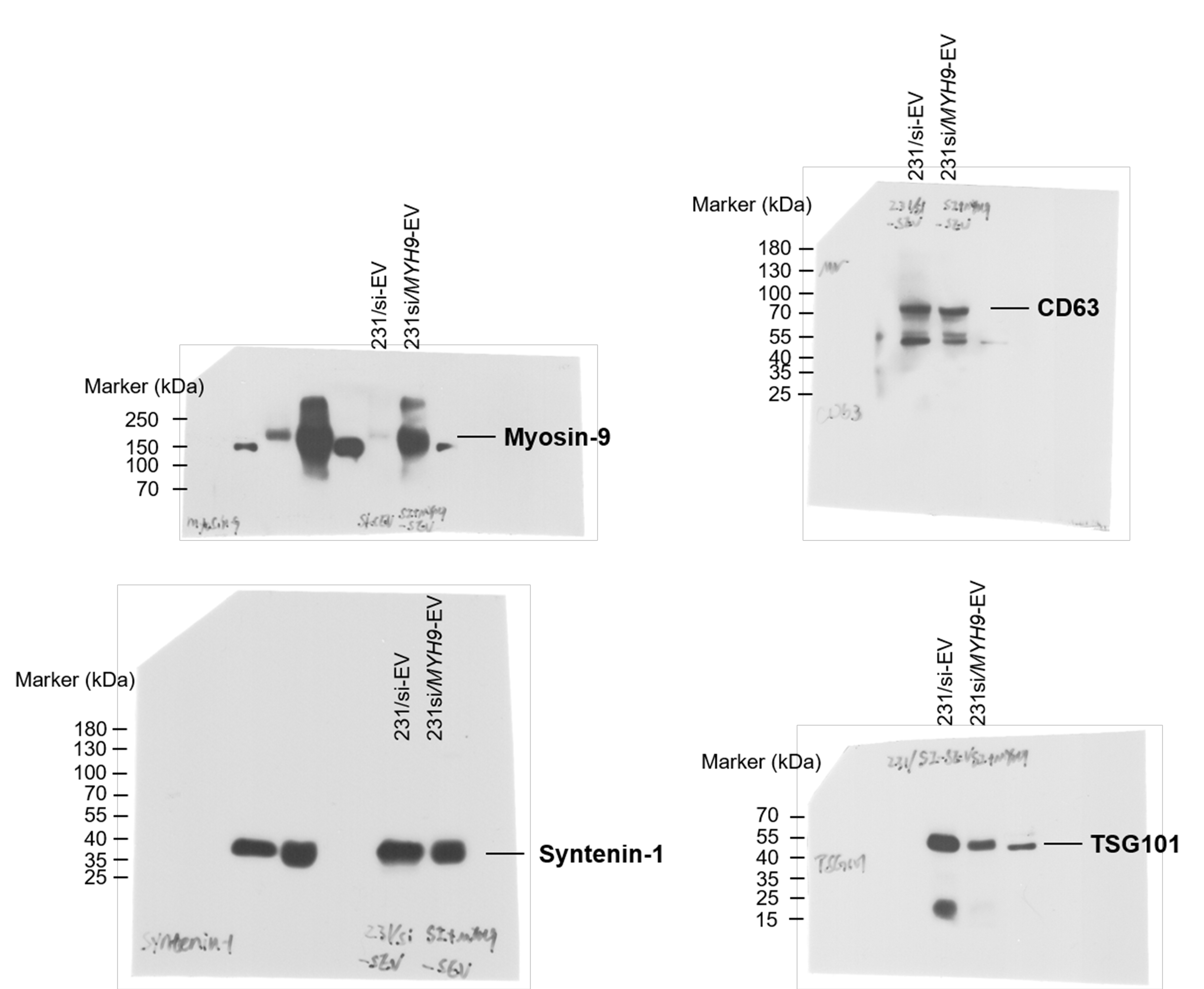

Supplement: Supplementary file 1 [file biology-11-00543-s001.zip › Original Images for western Blots and Gels/Figure 5C.tiff]

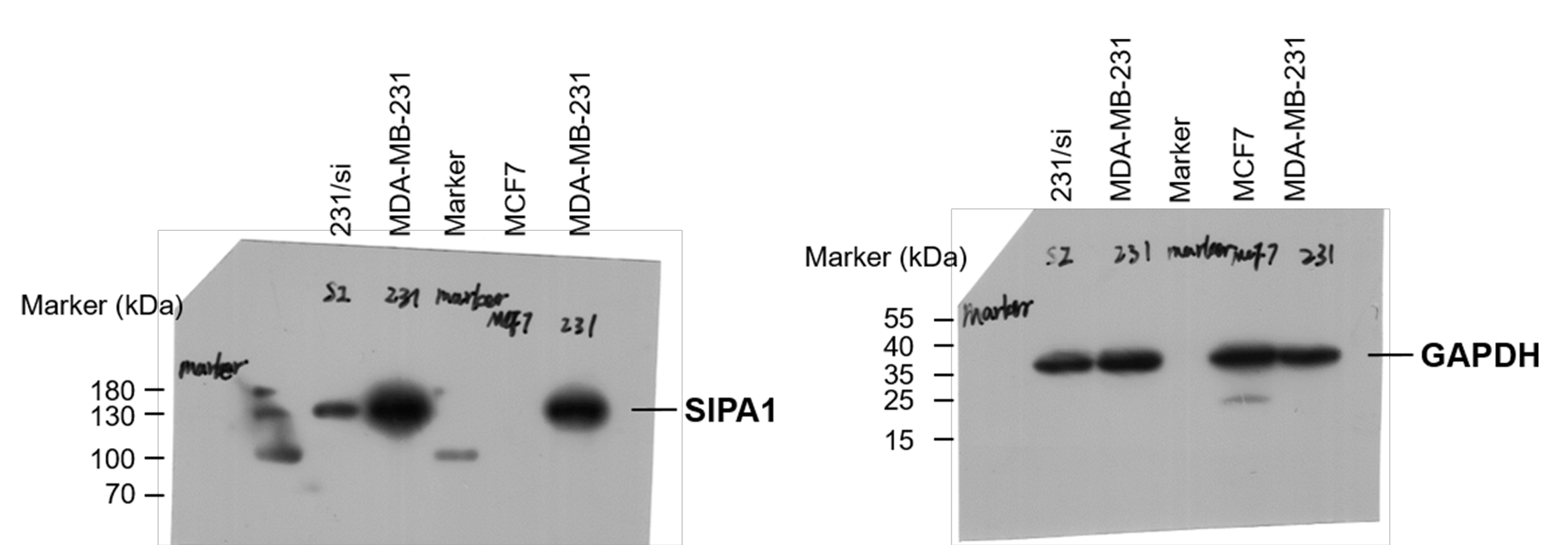

Supplement: Supplementary file 1 [file biology-11-00543-s001.zip › Original Images for western Blots and Gels/Figure S1A.tiff]

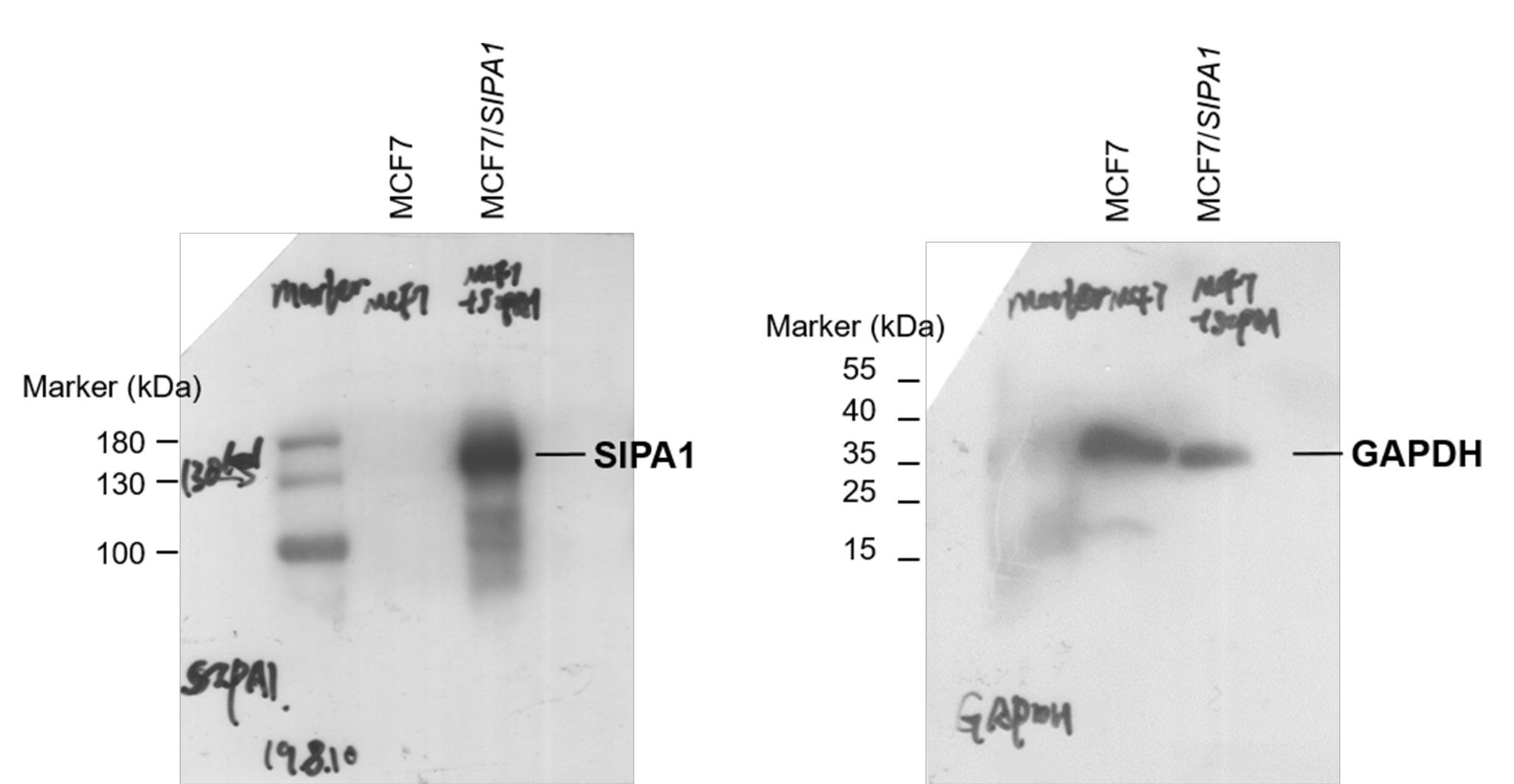

Supplement: Supplementary file 1 [file biology-11-00543-s001.zip › Original Images for western Blots and Gels/Figure S3A.tiff]

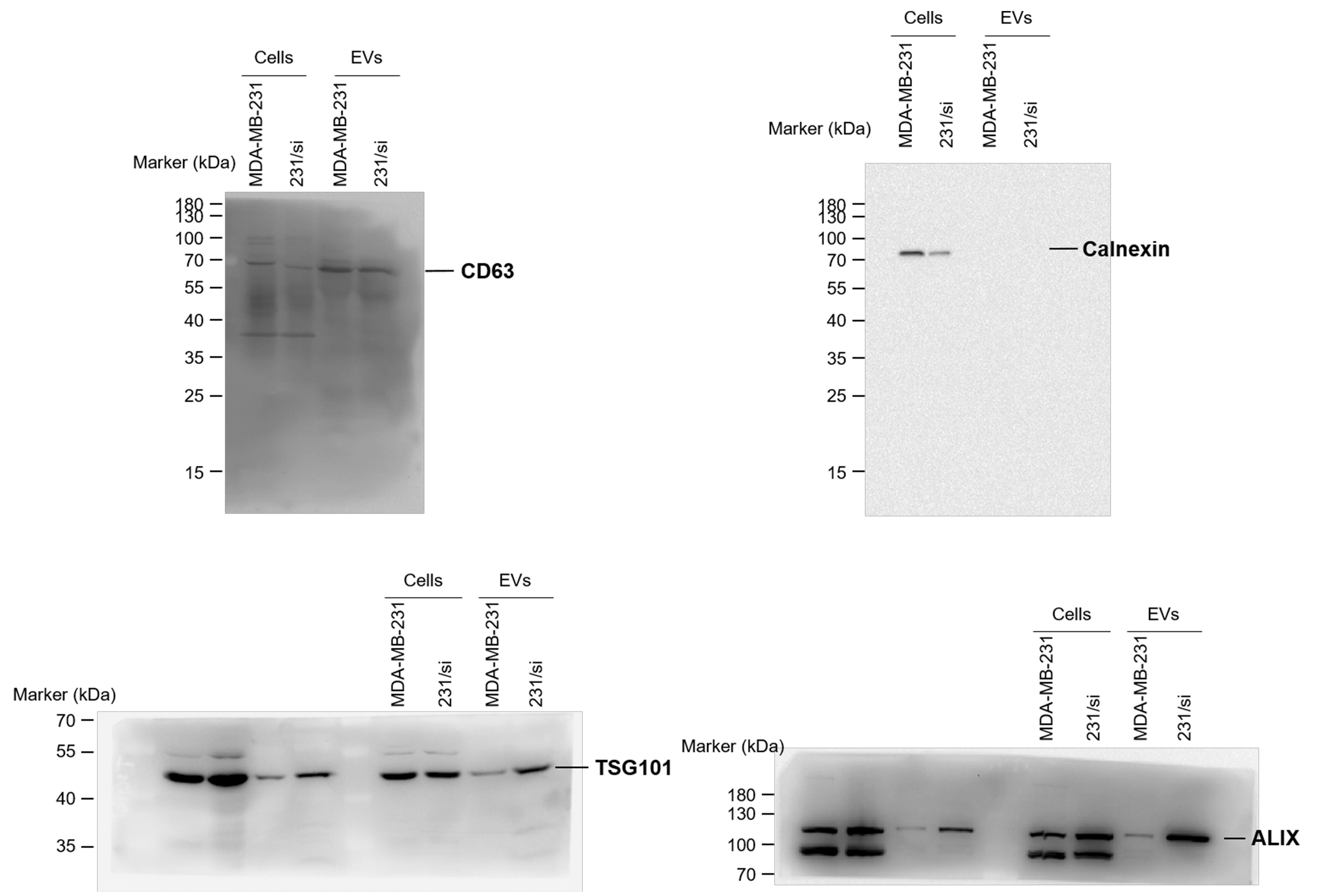

Supplement: Supplementary file 1 [file biology-11-00543-s001.zip › Original Images for western Blots and Gels/Figure S3B.tiff]

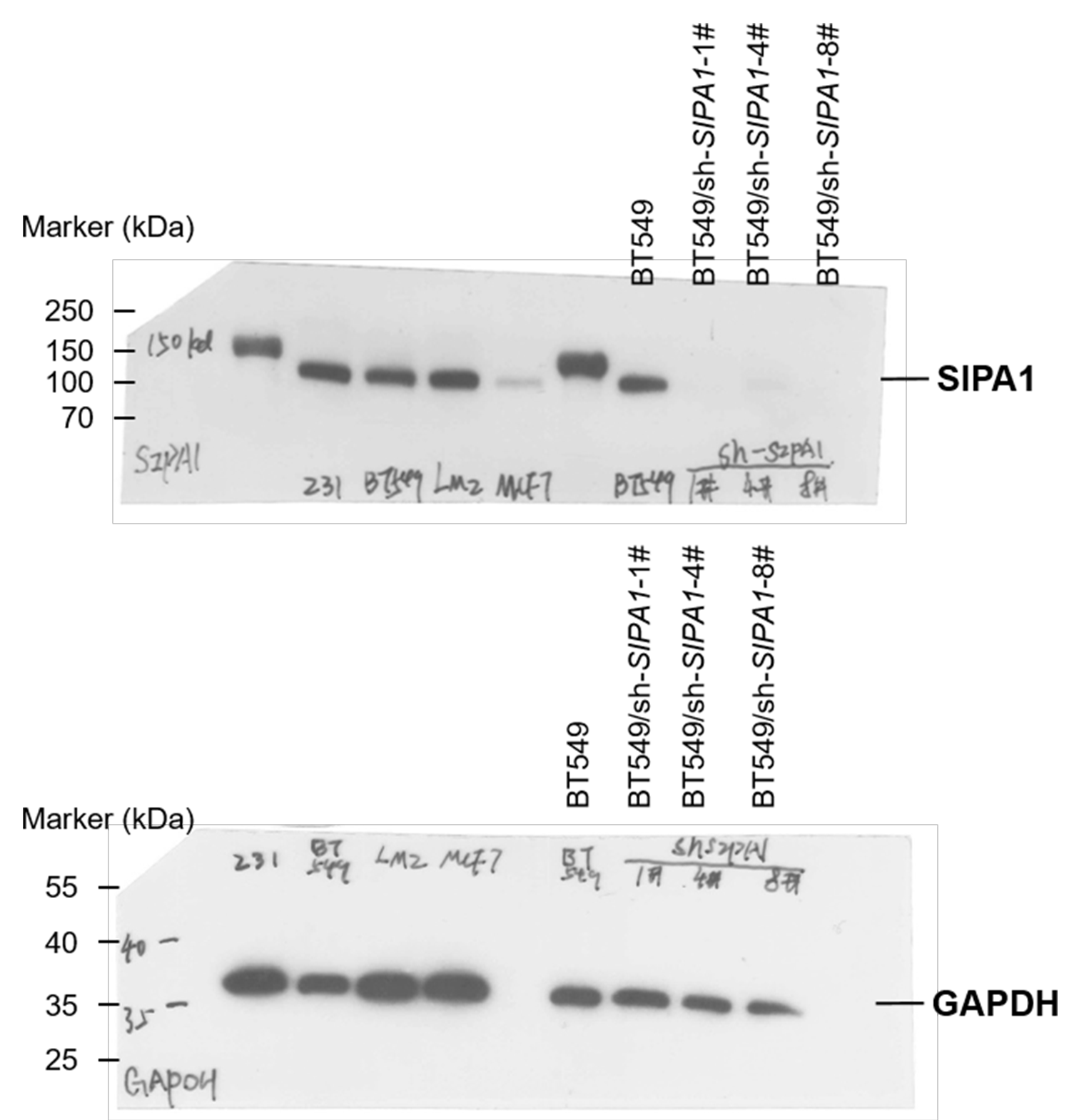

Supplement: Supplementary file 1 [file biology-11-00543-s001.zip › Original Images for western Blots and Gels/Figure S3F.tiff]

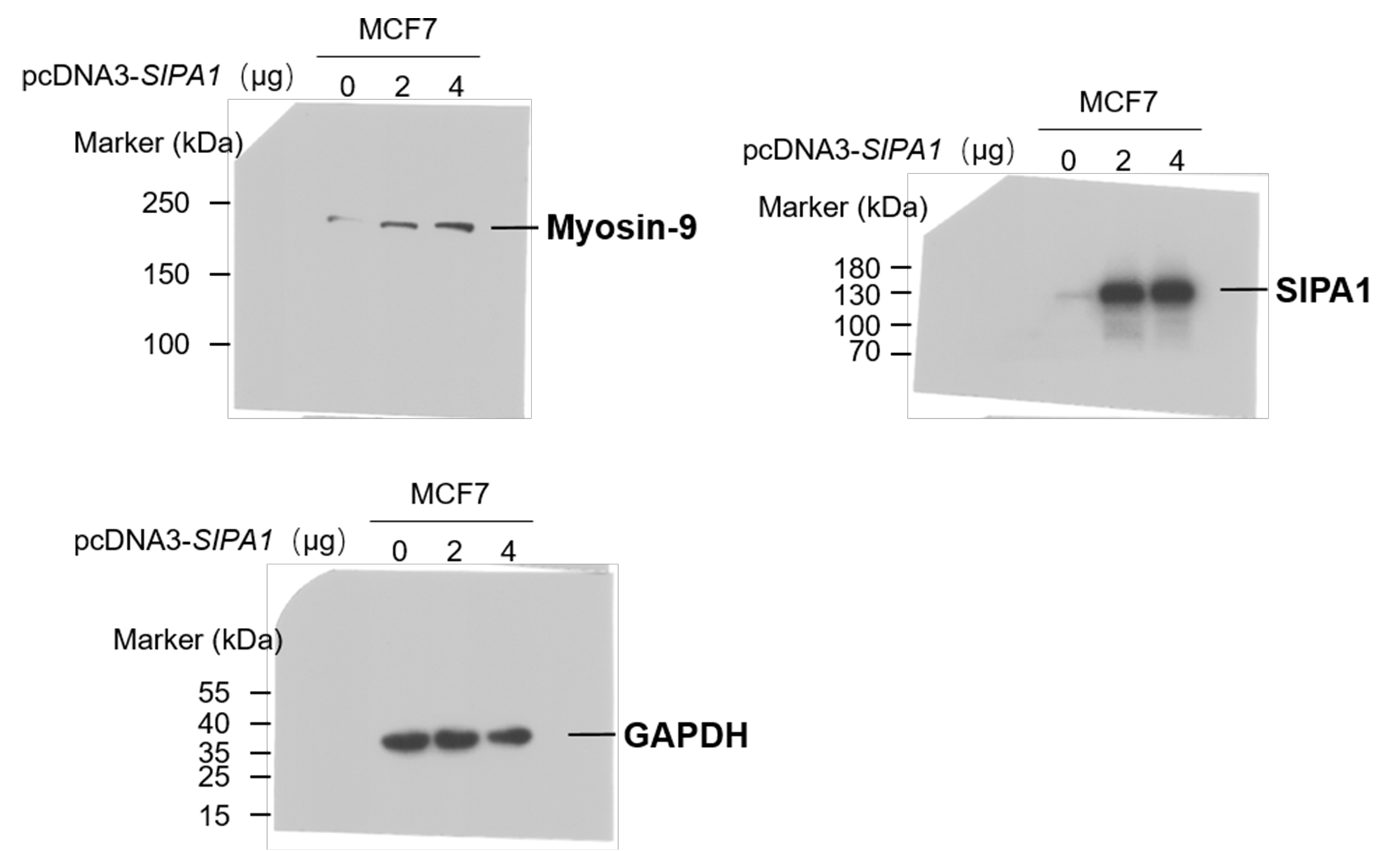

Supplement: Supplementary file 1 [file biology-11-00543-s001.zip › Original Images for western Blots and Gels/Figure S5B.tiff]

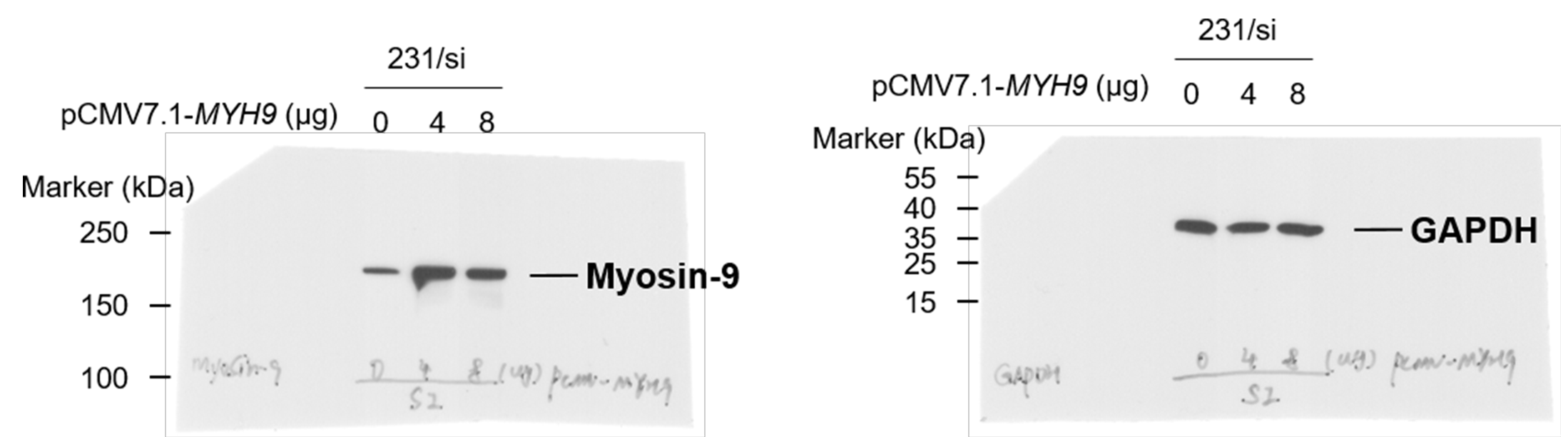

Supplement: Supplementary file 1 [file biology-11-00543-s001.zip › Original Images for western Blots and Gels/Figure S6A.tiff]

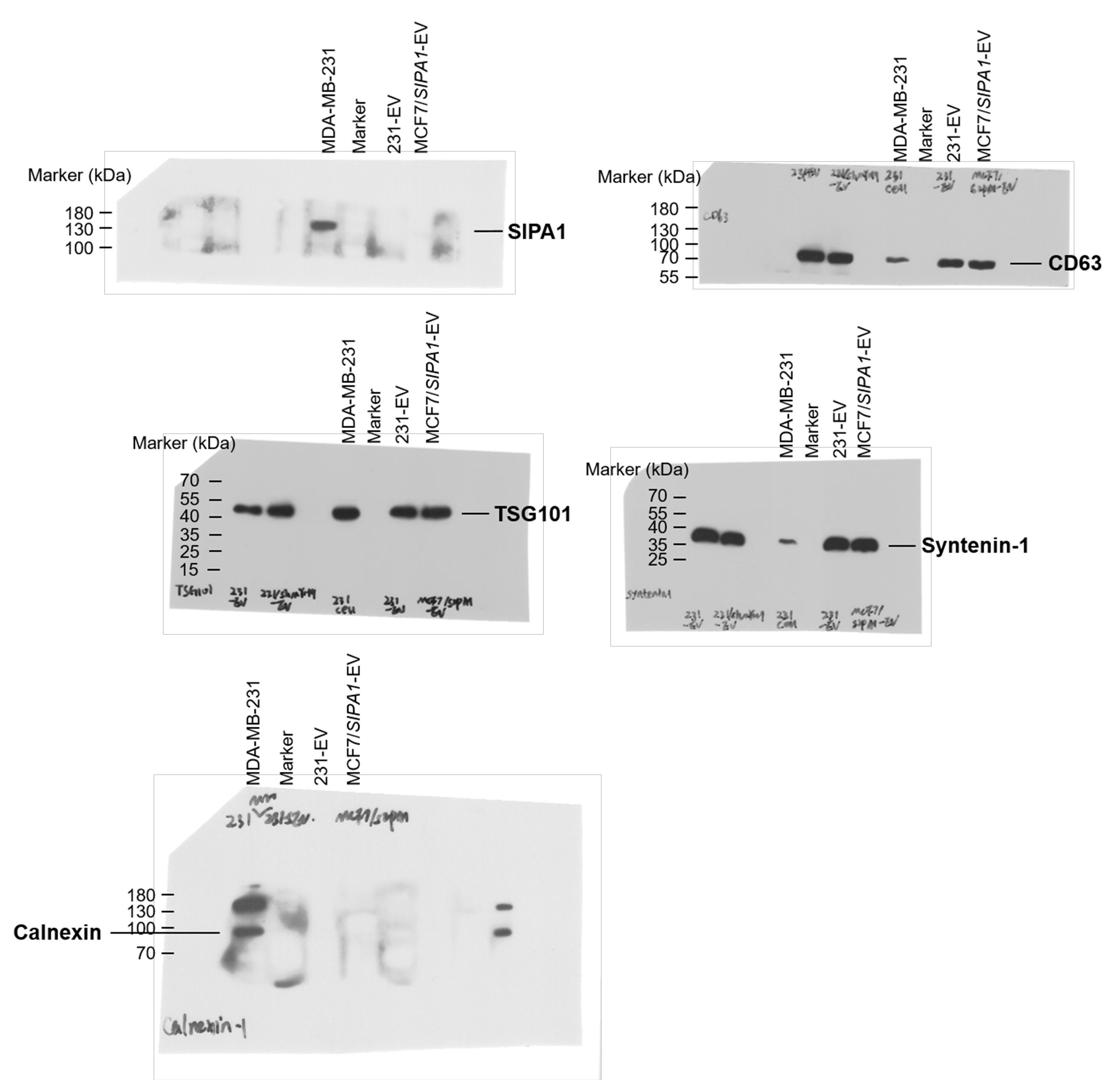

Supplement: Supplementary file 1 [file biology-11-00543-s001.zip › Original Images for western Blots and Gels/Figure S8A.tiff]
